# Global trends and health system impact on polycystic ovary syndrome: a comprehensive analysis of age-stratified females from 1990 to 2021

**DOI:** 10.3389/frph.2025.1642369

**Published:** 2025-10-23

**Authors:** Weiwei Zeng, Dali Gan, Juanfeng Ou, Brian Tomlinson

**Affiliations:** ^1^ Department of Pharmacy, Shenzhen Longgang Second People’s Hospital, Shenzhen, Guangdong, China; ^2^ Faculty of Medicine, Macau University of Science and Technology, Macao SAR, China

**Keywords:** female life-cycle prevalence, global burden of disease 2021, health system level, polycystic ovary syndrome, sociodemographic index

## Abstract

Polycystic ovary syndrome (PCOS) is a major factor in female infertility. The global burden of PCOS has generally been neglected due to insufficient data. This study aims to comprehensively assess the burden of PCOS among females aged 10–54 years globally from 1990 to 2021, to project trends for the next two decades, and to evaluate the impact of health system levels on PCOS prevalence. The incidence, prevalence, and disability-adjusted life years (DALYs) from the Global Burden of Disease (GBD) 2021 were analyzed, including global, sociodemographic index (SDI), GBD regions, health system levels, and national data. PCOS global burden escalated by 28% from 1990 to 2021. In 2021, the estimated annual percentage changes (EAPC) value of age-standardized rate of prevalence (ASPR), incidence, and DALYs were 0.77 (95% CI: 0.75–0.79), 0.74 (95% CI: 0.70–0.77), and 0.72 (95% CI: 0.68–0.75), respectively. High SDI regions showed elevated prevalence but slower growth (EAPC = 0.09) vs. low SDI regions, which exhibited the highest acceleration (EAPC = 1.23). Especially, the incidence and prevalence in the advanced health system were highest, but the most dramatic upward trend was observed in the basic level. The top three countries for ASPR were Italy (8,113.16 per 100,000 females, 95% CI: 5,757.74–11,265.85), Japan (6,334.11 per 100,000 females, 95% CI: 4,579.73–8,798.66), and New Zealand (5,689.13 per 100,000 females, 95% CI: 4,094.50–7,762.63). The incidence in adolescents aged 10–19 years was highest, warranting particular concern. This study underscored that health systems and adolescents require attention and strengthening as critical measures to control PCOS globally and to increase the fertility of women.

## Graphical Abstract

**Figure:**
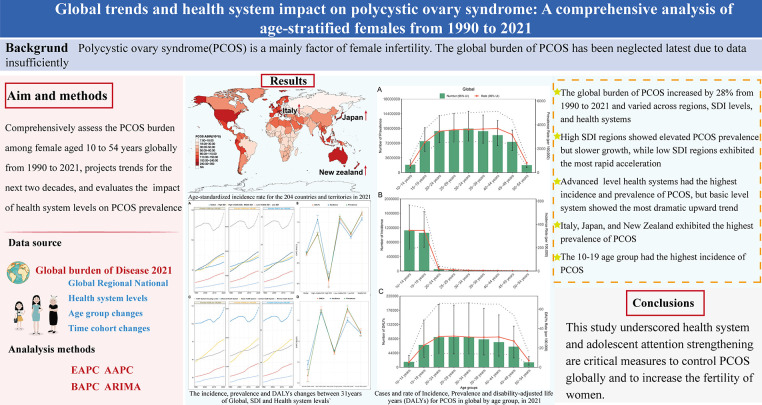

## 1 Introduction

Polycystic ovary syndrome (PCOS) is a chronic heterogeneous disorder characterized by irregular menstruation, infertility, obesity, and hyperandrogenism-related symptoms such as acne and hirsutism. Studies have shown that the global prevalence of PCOS is approximately 10%–13%, with slightly elevated rates observed in specific populations compared with others (1, 2). It plays an important role in female infertility. Beyond compromising reproductive health and diminishing quality of life in affected individuals, PCOS heightens the risk of long-term health complications, including cardiovascular diseases (e.g., hypertension, type 2 diabetes, and dyslipidemia), metabolic syndrome (MS), endometrial cancer (EC), and psychological conditions such as anxiety and depression (3, 4). These persistent health challenges significantly escalate healthcare expenditures, with global annual costs for PCOS management estimated at $7.9 billion by 2020 (5). While advancements in medical research have improved strategies for prevention, diagnosis, and treatment of PCOS, the syndrome continues to impose substantial health, societal, and economic burdens worldwide. A comprehensive assessment of its current global impact remains critically important to inform targeted interventions and policy development. Current PCOS treatment remains limited to lifestyle modifications (e.g., physical exercise) and pharmacological interventions targeting comorbidities, including hormonal medications, insulin sensitizers, and lipid-lowering agents (6, 7). To address therapeutic gaps and reduce healthcare costs, preclinical research has identified natural compounds with potential efficacy, such as plant-derived polysaccharides and bioactive extracts (8–10).

The Global Burden of Disease (GBD) database 2021 is a platform containing standardized health data that quantifies the burden and societal impact of 371 diseases and 88 risk factors, among 204 countries and territories over the past 31 years (11, 12). PCOS remains one of the studied diseases within this framework. Prior epidemiological investigations employing Global Burden of Disease datasets have systematically characterized PCOS burden patterns. A multinational analysis spanning 194 countries and regions revealed progressive increases in age-standardized incidence rates (ASIR) and disability-adjusted life years (DALYs) metrics associated with PCOS from 2007 to 2017 (13). Complementary regional assessments focusing on Middle Eastern and North African region populations demonstrated substantial burden escalation between 1990 and 2019, with age-standardized incidence climbing 33.7%, prevalence rising 37.9%, and years lived with disability (YLDs) increasing 36.1% across these territories. At the national level, the analysis demonstrated a positive correlation between age-standardized YLDs and the sociodemographic index (SDI) (14). Global analysis demonstrated PCOS burden across 204 countries year-on-year increased in almost all countries from 1990 to 2019, with differences in incidence, prevalence, and YLDs between countries, with predictive models indicating sustained upward trajectories over the subsequent two decades (15–17). While most of these findings confirm an escalating global trend in PCOS burden over time and highlight enduring disparities across geographical regions, national boundaries, and socioeconomic populations, current conclusions remain constrained by some limitations. Existing evidence primarily derives from regional studies and historical data modeling frameworks. Crucially, no comprehensive assessment has yet leveraged the updated GBD 2021 dataset to perform a contemporary global evaluation and analysis of PCOS burden.

To bridge this knowledge gap, we conducted an updated assessment of PCOS burden trends from 1990 to 2021 across global, regional, and national levels, including age-standardized incidence, prevalence, and DALYs. Given that the burden of PCOS predominantly manifests through health complications and quality-of-life impairments, with minimal likelihood of premature mortality attributable to the condition, DALYs in this study are equivalent to YLDs (11). We presented PCOS burden variations across countries and regions stratified by SDI and healthcare system characteristics. In addition, age-specific analyses were performed to identify populations most affected by PCOS and to inform targeted prevention and treatment strategies. This current GBD study on PCOS not only facilitates optimized allocation of healthcare resources and supports the development of precision public health policies across diverse regions but also elevates public awareness of PCOS and strengthens individual consciousness of self-directed intervention strategies.

## 2 Methods

PCOS is a common endocrine and metabolic abnormality of reproduction in women, with the main features including sparse ovulation or anovulation, polycystic ovarian morphology, hyperandrogenism, or related clinical manifestations (e.g., hirsutism and acne). Hyperandrogenism and ovulatory dysfunction are the core features of the current three diagnostic criteria of PCOS. All of them emphasize the exclusion of other conditions that may cause similar manifestations, such as congenital adrenal hyperplasia (CAH), thyroid dysfunction, and hyperprolactinemia. The PCOS in GBD 2021 was defined by self-report and a doctor's diagnosis with one of three criteria, including National Institutes of Health (NIH) (1990), Rotterdam (2003), or AE-PCOS (2006), which were standardized in GBD 2021 and are based on the definition set forth by the American College of Obstetricians and Gynecologists. The data reports followed the program's guidelines to ensure data accuracy. PCOS cases were identified by the International Classification of Diseases (ICD) code 10, E28.2 (18). For further details, refer to the previous study (19).

### 2.1 Data sources

Data from GBD 2021 could be obtained from https://vizhub.healthdata.org/gbd-results/. As an updated version of the GBD 2019 database, it provides comprehensive global epidemiological estimates for 371 diseases and injuries, including population data, mortality, and DALYs. The detailed information on the design and methodology of the GBD 2021 database study has been extensively described in the previous research (19). Input data were derived from multiple sources, including disease registries and patient medical records, and underwent rigorous standardization to ensure consistency and completeness. All estimates are accompanied by 95% uncertainty intervals (UIs), reflecting potential variations and data gaps. Notably, since the GBD 2021 does not include risk factor assessments specific to PCOS, this aspect was beyond the scope of the present study.

All regional, age, and class divisions were developed by the Institute for Health Metrics and Evaluation (IHME), and the regions are indicative only of the geographic areas used for the subgroup analyses and do not relate to geopolitical units. SDI is a comprehensive index of a country's or region's state of development. The closer the value is to 1, the better the socioeconomic development of the region or country. The project applies the SDI value to classify the global into five regions. In particular, the project categorizes the level of health systems across all the regions and countries in the GBD 2021 database into four levels (advanced, basic, limited, and minimal). The diagnosis and screening of PCOS may influence the distribution of PCOS burden, due to the medical level of the four-level health system. The health system data could be used to analyze the impact of different levels of health systems on the PCOS disease burden.

This study used the global, regional, and national data from 1990 to 2021, which included the incidence, prevalence, and DALYs' number and rate of PCOS females of all ages, age-standardized, and between 10 and 54 years of age (in groups of 5 years each). The 95% CI of all data was recorded.

The incidence, prevalence, DALYs, YLDs, and years of life lost (YLL) to premature death are the core metrics in GBD research. The DALYs are the total of YLDs and YLL, but there is no evidence of death due to PCOS. This is because the burden of PCOS is mainly in the form of long-term health problems and reduced quality of life due to complications such as hyperandrogenemia and infertility, with a low death risk.

### 2.2 Statistical analysis

The estimated annual percentage change (EAPC) of the age-standardized rate (ASR) in females was calculated using the data from the GBD 2021 database between 1990 and 2021. These estimates were used to compare the effects of different factors on the trends in PCOS incidence, prevalence, and DALYs. The data were prepared and calculated as previously described (20). Briefly, a generalized linear model with a Gaussian distribution was fit to the natural logarithm of ASR values, and the EAPC quantified the temporal trend in ASR for PCOS disease between 1990 and 2021, along with its 95% CI. When the EAPC values and the upper limit of its 95% CI are negative, a decreasing trend in ASR for PCOS was indicated, whereas an increasing trend if the opposite is true. When the 95% CI contained 0, the ASR was considered to be stable within the specified time frame. In addition, the trend of its segments was calculated using the software Joinpoint (version 5.4.0).

### 2.3 Bayesian age–period–cohort and autoregressive integrated moving average projection

Autoregressive integrated moving average (ARIMA), a time series forecasting method, was selected for its robust performance in analyzing temporal dependence, trends, and forecasting of epidemiological data. The model is characterized by three parameters: *p* (autoregressive order), *d* (degree of differencing), and *q* (moving average order). Prior to model fitting, stationarity was rigorously assessed using the augmented Dickey–Fuller (ADF) test, with the minimal differencing order (*d*) determined when the ADF *p*-value fell below 0.05. After confirming stationarity of the PCOS ASR time series, candidate models were automatically fitted using the R package “forecast.” The optimal model was selected by minimizing the corrected Akaike information criterion (AIC) and Bayesian information criterion (BIC), followed by validation through the Shapiro–Wilk normality test and Ljung–Box white noise test to ensure residual independence and normality. Autocorrelation and partial autocorrelation functions were examined to detect any remaining temporal dependence in the residuals. Finally, forecasts were generated for the period 2022–2042 and visualized using the “t.series” package, following a previously described methodology (11).

The Bayesian age–period–cohort (BAPC) model was also employed for the prediction of disease prevalence. While ARIMA forecasts the aggregate time series, it explicitly disentangles the effects of age (A), time period (P), and birth cohort (C). This is essential for identifying whether changes in burden are due to factors affecting all ages equally in a given year (period effect) or specific generations (cohort effect). And they allow for the integration of projected demographic data to calculate future age-specific rates and case counts. It facilitates the analysis of trends in age and time series cohorts by leveraging projected demographic data and the substitution of standardized age structures. BAPC models that include nested Laplace approximation can provide more diverse models and fit with higher accuracy and wider coverage. BAPC and INLA R packages were used for the projection (21). The Integrated Nested Laplace Approximation (INLA) algorithm was chosen for its computational efficiency and accuracy in approximating Bayesian posterior distributions compared with traditional Markov chain Monte Carlo (MCMC) methods. Model selection and fit were evaluated using the deviance information criterion (DIC), Watanabe–Akaike information criterion (WAIC), and continuous ranked probability score (CRPS). Furthermore, a sensitivity analysis was conducted by varying the hyperparameters of the random walk priors governing the smoothness of the period and cohort effects.

All statistical analyses and data visualization were performed using RStudio (version 4.4.1), and *P* < 0.05 was considered statistically different. R packages (“ggplot2”, “ggmap,” “patchwork,” “EAPC,” “BAPC,” “ARIMA,” “forecast,” “tseries,” and “data.table”) were used for data computation and graphing.

## 3 Results

### 3.1 Global, regional, and national trends of PCOS from 1990 to 2021

#### 3.1.1 Global trends

**Figure 1:**
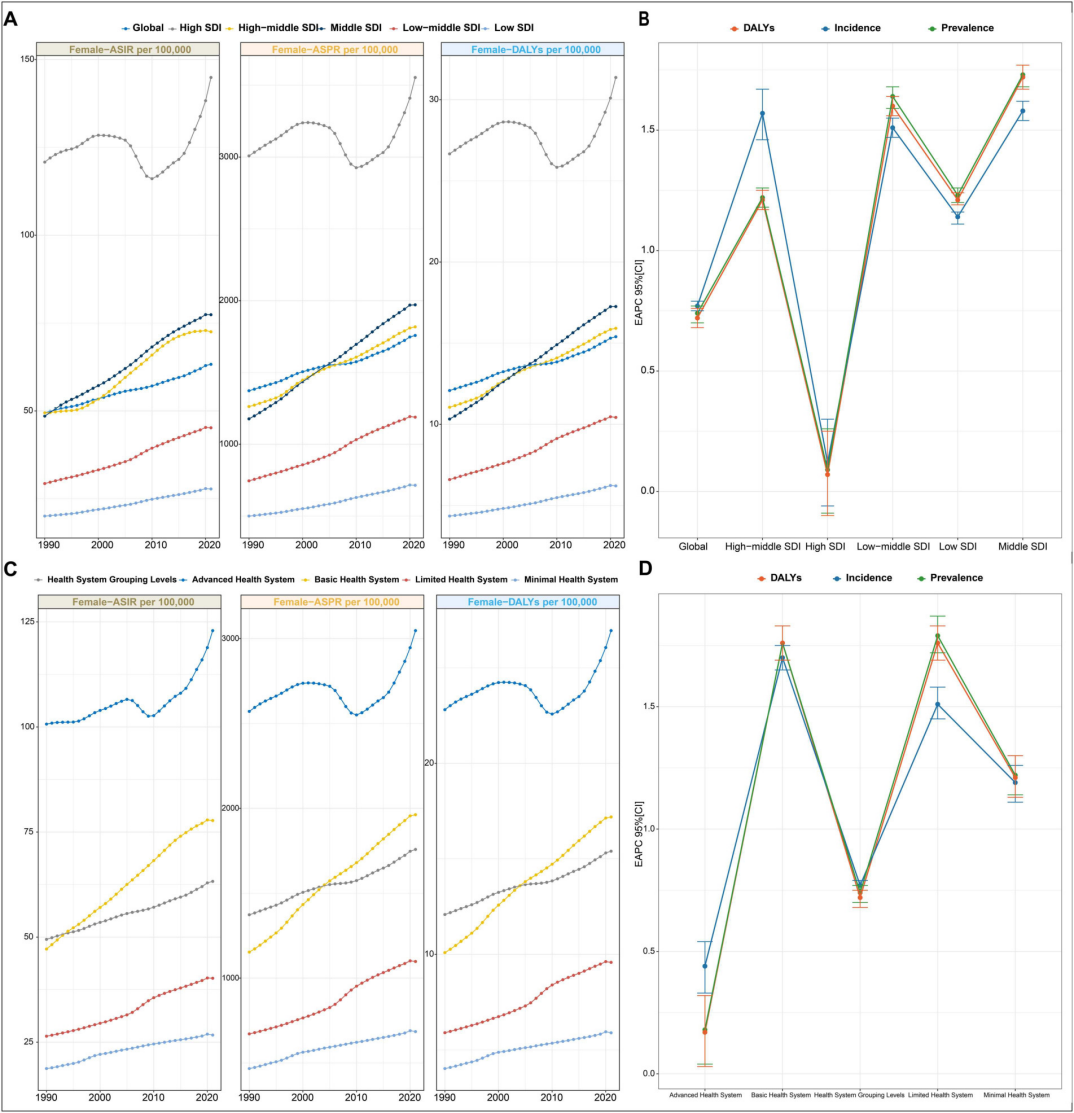
PCOS burden in different regions from 1990 to 2021. (**A**) Trends in ASPR, ASIR, and ASDR for PCOS at the global level and by SDI regions from 1990 to 2019. Values are shown on the left *y*-axis in per 100,000; year is indicated on the bottom *x*-axis. (**B**) EAPC in incidence, prevalence, and DALYs for PCOS, stratified by SDI quintiles. (**C**) Trends in ASPR, ASIR, and ASDR for PCOS in different health system regions from 1990 to 2019. Metrics are presented as rates per 100,000 females (left *y*-axis); the bottom *x*-axis denotes the year. (**D**) EAPC in incidence, prevalence, and DALYs for PCOS, categorized by health system level. EAPC, estimated annual percentage change; ASPR, age-standardized prevalence rate; ASIR, age-standardized incidence rate; ASDR, age-standardized DALY rate; SDI, sociodemographic index.

The number of patients with PCOS is rising at a significant rate globally, reaching 69,473,252.37 cases (95% CI: 49,531,420.00–95,724,479.23) by 2021, which is an exaggerated 47-fold increase from 1,476,225.27 cases (95% CI: 1,057,983.5–2,045,276.94) in 1990. The global age-standardized prevalence rate (ASPR) of PCOS was 1,757.83 per 100,000 females, an increase of 28.05% from 1,372.77 in 1990. And the incidence of PCOS is on the rise globally. ASIR of PCOS increased from 49.45 per 100,000 females in 1990 to 63.26 in 2021, which is a 27.93% growth rate. In 2021, the global DALYs for PCOS were 607,756.87 cases (95% CI: 272,745.15–1,268,607.22), with an age-standardized rate of DALYs (ASDR) increasing from 12.08 per 100,000 females in 1990 to 15.4 in 2021, an increase of 27.48%. EAPC of age-standard global PCOS incidence, prevalence, and DALYs were 0.77 (95% CI: 0.75–0.79), 0.74 (95% CI: 0.70–0.77), and 0.72 (95% CI: 0.68–0.75), respectively, which reflect an overall increasing trend of PCOS patients in the worldwide (Table 1, Figure 1A).

**Table 1:** Global and regional trends in PCOS burden: incidence and EAPC from 1990 to 2021.

Location	Number_1990	ASR_1990	Number_2021	ASR_2021	EAPC_CI
Global	14,76,225.27 (10,57,983.5–20,45,276.94)	49.45 (35.57–68.45)	23,01,505.64 (16,55,989.24–31,67,177.81)	63.26 (45.41–87.28)	0.77 (0.75–0.79)
SDI					
High SDI	437,301.50 (319,330.75–606,631.95)	120.81 (88.32–167.22)	494,212.24 (367,071.51–670,948.04)	144.89 (107.28–196.81)	0.12(−0.06 to 0.30)
High-middle SDI	261,030.54 (187,118.65–358,760.32)	49.47 (35.43–68.6)	305,809.98 (216,061.40–424,907.89)	72.52 (51.14–101.53)	1.57 (1.47–1.67)
Middle SDI	505,653.92 (359,587.99–703,114.26)	48.52 (34.54–67.44)	812,668.96 (572,963.70–11,26,099.74)	77.38 (54.44–107.63)	1.58 (1.54–1.62)
Low-middle SDI	209,333.49 (148,000.10–293,837.26)	29.32 (21.09–40.77)	481,689.01 (338,285.68–670,310.33)	45.20 (31.82–62.95)	1.51 (1.47–1.55)
Low SDI	61,881.63 (43,355.56–87,726.03)	20.08 (14.45–28.07)	205,458.30 (144,223.85–291,379.81)	27.79 (19.81–38.87)	1.14 (1.11–1.16)
Health system level					
Advanced health system	567,808.60 (414,557.75–781,167.82)	100.68 (73.42–139.19)	608,234.01 (449,376.29–823,362.31)	122.92 (90.46–165.99)	0.44 (0.33–0.54)
Basic health system	640,190.39 (455,028.48–889,468.88)	47.16 (33.41–65.57)	961,899.91 (678,292.02–13,40,880.52)	77.73 (54.68–108.66)	1.70 (1.65–1.75)
Limited health system	2,51,237.22 (1,77,968.55–3,52,944.48)	26.42 (19.14–36.69)	6,69,132.19 (4,77,165.34–9,30,045.33)	40.21 (28.62–55.9)	1.51 (1.45–1.57)
Minimal health system	15,964.87 (11,131.54–22,579.74)	18.72 (13.33–26.08)	60,572.37 (42,401.67–85,645.52)	26.69 (18.93–37.48)	1.19 (1.12–1.26)
Regions					
Andean Latin America	25,250.91 (17,294.54–36,023.12)	100.30 (68.88–142.65)	42,634.55 (29,473.44–60,429.45)	134.14 (92.65–190.7)	0.90 (0.81–0.99)
Australasia	15,948.65 (11,745.49–20,802.41)	173.96 (127.23–229.44)	21,492.87 (15,427.88–29,606.77)	202.25 (145.07–278.86)	0.27 (0.18–0.36)
Caribbean	9,226.66 (6,288.13–13,042.76)	44.33 (30.33–62.54)	11,962.48 (8,144.39–16,730.14)	55.61 (37.61–78.19)	0.81 (0.75–0.88)
Central Asia	5,347.79 (3,686.14–7,702.43)	13.68 (9.46–19.63)	8,122.32 (5,654.13–11,355.50)	18.79 (13.06–26.29)	1.13 (1.06–1.20)
Central Europe	4,244.91 (2,905.82–6,223.94)	7.35 (5.01–10.81)	3,175.48 (2,221.05–4,441.04)	8.97 (6.22–12.67)	0.57 (0.50–0.63)
Central Latin America	1,19,114.89 (81,948.23–1,68,844.63)	107.23 (73.94–151.71)	1,43,013.73 (1,00,629.38–2,00,290.73)	115.89 (81.09–162.25)	−0.11 (−0.28 to 0.05)
Central Sub-Saharan Africa	6,063.01 (4,247.42–8,685.30)	17.63 (12.55–24.87)	23,871.94 (16,637.67–33,976.02)	26.20 (18.46–36.97)	1.22 (1.08–1.36)
East Asia	2,18,485.79 (1,54,343.69–3,01,132.94)	32.14 (22.85–44.87)	2,68,458.51 (1,90,157.13–3,73,877.35)	58.63 (41.2–82.32)	1.97 (1.83–2.11)
Eastern Europe	8,328.34 (5,913.32–11,639.17)	8.56 (5.98–12.09)	7,596.11 (5,457.51–10,592.63)	10.86 (7.64–15.38)	0.93 (0.88–0.97)
Eastern Sub-Saharan Africa	25,901.96 (18,214.02–36,986.15)	20.71 (14.82–29.05)	76,605.14 (53,945.13–1,08,540.53)	26.26 (18.57–36.96)	0.82 (0.79–0.86)
High-income Asia Pacific	1,58,931.18 (1,11,210.64–2,26,424.22)	193.02 (137.42–268.99)	1,07,170.54 (75,693.68–1,52,114.34)	225.11 (160.59–317.34)	0.40 (0.33–0.48)
High-income North America	1,34,112.71 (94,940.5–1,87,144.22)	119.24 (83.99–166.82)	2,03,413.49 (1,50,004.56–2,71,137.80)	149.79 (111.27–197.64)	−0.56 (−1.04 to 0.08)
North Africa and Middle East	1,28,593.52 (88,937.33–1,83,308.60)	59.01 (41.15–83.29)	2,42,427.05 (1,71,058.09–3,42,784.62)	77.15 (54.45–109.17)	0.96 (0.92–1.00)
Oceania	2,013.15 (1,401.24–2,847.04)	49.56 (34.79–69.76)	5,300.91 (3,692.06–7,439.78)	69.33 (48.42–97.52)	0.85 (0.69–1.01)
South Asia	1,62,363.29 (1,16,293.11–2,26,382.69)	25.33 (18.35–34.97)	4,16,258.43 (2,99,377.4–5,73,874.15)	42.08 (29.94–58.32)	1.88 (1.80–1.96)
Southeast Asia	1,74,333.20 (1,22,499.01–2,43,431.39)	58.95 (41.50–82.07)	3,57,262.30 (2,54,498.65–4,95,461.45)	110.11 (77.87–153.89)	2.32 (2.22–2.42)
Southern Latin America	12,429.77 (8,602.22–17,727.21)	46.78 (32.37–66.78)	21,574.43 (15,329.63–30,970.01)	74.53 (52.77–106.57)	1.46 (1.24–1.67)
Southern sub-Saharan Africa	11,799.72 (8,261.02–16,787.82)	33.79 (23.76–47.64)	18,377.82 (12,888.44–26,037.99)	42.33 (29.66–59.97)	0.73 (0.65–0.82)
Tropical Latin America	22,284.58 (15,199.83–32,003.99)	23.31 (15.97–33.28)	23,769.11 (16,611.45–32,960.09)	24.90 (17.12–35.01)	−0.22 (−0.4 to 0.05)
Western Europe	2,06,740.90 (1,46,330.87–2,86,172.09)	143.35 (100.92–200.55)	2,06,192.21 (1,45,653.51–2,86,689.08)	154.64 (109.19–215.84)	0.15 (0.11–0.19)
Western sub-Saharan Africa	24,710.33 (17,219.71–35,159.50)	20.04 (14.4–27.97)	92,826.23 (65,226.48–1,32,560.86)	27.80 (19.81–39.19)	0.83(0.66–0.99)

#### 3.1.2 Regional level

##### 3.1.2.1 Regional trends

In 2021, the burden of PCOS showed significant geographic variation, with the five regions having the highest ASIR including high-income Asia Pacific, Australasia, Western Europe, Western Europe high-income North America, and Andean Latin America. Interestingly, among the EAPC results of these five regions, only high-income North America showed a downward trend in incidence [EAPC: −0.56 (95% CI: −1.04 to −0.08)]. The Andean Latin America exhibited the most significant upward trend [EAPC: 0.90 (95% CI: 0.81–0.99)]. Comparatively, Central Europe, Eastern Europe, Central Asia, Tropical Latin America, and Central Sub-Saharan Africa had the lowest ASIR. In particular, Tropical Latin America was the only region among these five regions that showed negative growth [EAPC: −0.22 (95% CI: −0.4 to −0.05)], while Central Sub-Saharan Africa is the fastest growing region, with an EAPC of 1.22 (95% CI: 1.08–1.36). Among all regions, Southeast Asia had the highest EAPC value of [2.32 (95% CI: 2.22–2.42)] for incidence rate, followed by East Asia and South Asia. These data revealed a significant increasing trend in the prevalence of PCOS in Asian populations. In contrast to the high ASIR, high-income North America had the lowest EAPC value of incidence rate, which was −0.56 (95% CI: −1.04 to −0.08) and a negative trend, suggesting that the incidence of patients with PCOS is slowly decreasing. Regionally, only Tropical Latin America [EAPC −0.22 (95% CI: −0.4 to −0.05)] showed a decreasing trend (Table 1). The ASPR change of PCOS in high-income Asia Pacific was consistent with that in ASIR. The Andean Latin America had the most pronounced increase, with an EAPC of 1.08 (95% CI: 1.0–1.05). And the five lowest ASPR regions were seen as in ASIR. Tropical Latin America, with an EAPC of −0.14 (95% CI: −0.3 to 0.02), showed a generally stable trend as its EAPC CI spans 0, whereas the other four regions all exhibited an upward trend. And Central Asia had the highest increase, with an EAPC of 1.18 (95% CI: 1.11–1.25). For detailed information on regional trends in ASPR and ASDR changes, see Supplementary Table S1.

##### 3.1.2.2 SDI regional trends

ASPR, ASIR, and ASDR exhibited distinct temporal trajectories across the five SDI quintiles from 1990 to 2021. Consistently across the study period, high SDI quintiles maintained substantially elevated ASPR, ASIR, and ASDR. Its temporal evolution exhibited a triphasic trajectory characterized by an initial ascent (1990–2005), followed by a transient decline (2005–2015), and a subsequent resurgence (2015–2021). A notable reversal emerged post-2010, with these metrics demonstrating sustained progressive increases, culminating in 2021, the ASIR values of 144.89 per 100,000 females (95% CI: 107.2–196.81). This pattern resulted in EAPC with 95% CI straddling 0 for ASIR, ASPR, and ASDR of the high SDI region, collectively indicating statistically non-significant trend directions over the three-decade span (Figure 1A and Table 1). For instance, the most pronounced growth occurred in the middle SDI group. Geospatial analysis revealed predominantly positive EAPC in Prevalence across all the regions, with the middle SDI group demonstrating the highest EAPC value and the high SDI group displaying the most pronounced elevation again. This pattern paralleled the ASR of DALY trajectories (Figure 1B).

##### 3.1.2.3 Health system level trends

Stratified by health system group levels, the minimal health system exhibited the lowest ASPR, ASIR, and ASDR in both 1990 and 2021 (Table 1). However, the advanced health system consistently demonstrated the highest values across all three metrics during the same observation period (Figure 1C).

In 2021, the advanced health system group uniquely demonstrated a negative EAPC value for ASPR, ASIR, and ASDR. Conversely, minimal, limited, and basic health system groups exhibited positive EAPC values across all three metrics. Among these, the limited health system group showed the most pronounced elevation in EAPC value of ASPR and ASDR, while the basic health system group had the highest EAPC value of ASIR (Figure 1D).

#### 3.1.3 National trend

**Figure 2:**
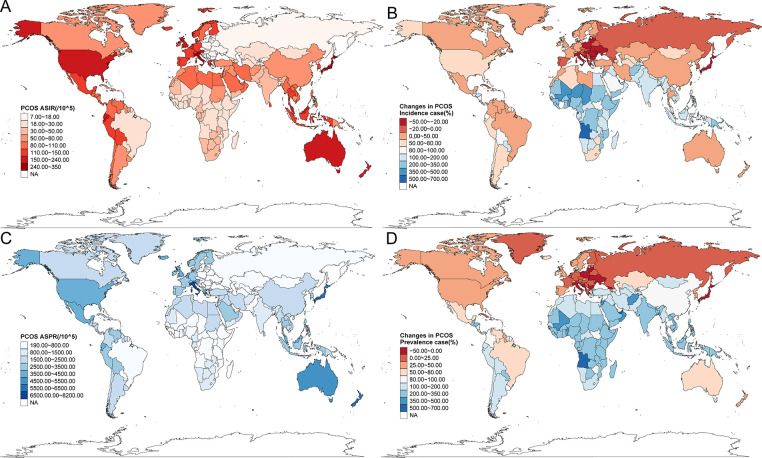
The disease burden of PCOS in 204 countries and territories in 2021. (**A**) ASIR for the 204 countries. (**B**) The changes in PCOS incidence cases from 1990 to 2021. (**C**) ASPR for the 204 countries. (**D**) The changes in PCOS prevalence cases from 1990 to 2021.

The GBD database encompassed epidemiological data on PCOS from 204 countries and territories worldwide. All the countries' PCOS situation revealed substantial geographic heterogeneity. ASIR varied globally from 7 to 350 cases per 100,000 females, and ASPR ranged from 190.00 to 8,200.00 per 100,000 females. Italy, Japan, New Zealand, Australia, and Malaysia consistently exhibited the highest ASIR and ASPR values. In contrast, several countries in Southeast Europe, including Bosnia and Herzegovina, Albania, North Macedonia, and Serbia, demonstrated the lowest rates, with ASIR below 8 per 100,000. Notably, the United States, the United Kingdom, and Austria also ranked among the top-tier nations for PCOS incidence (Figures 2A,C; Supplementary Table S3). Between 1990 and 2021, Equatorial Guinea demonstrated the most substantial incidence increase (684.19%), followed by Angola (516.20%), Benin (453.77%), and Qatar (404.31%). In contrast, the steepest declines were concentrated in Central, Southern, and Eastern Europe, even including Japan in Asia, with the United States Virgin Islands (−39.39%), Japan (−35.79%), Albania (−34.34%), Italy (−32.75%), and Poland (−32.04%) showing the most significant reductions (Figure 2B). As shown in Figure 2D and Supplementary Table S2, between 1990 and 2021, the most pronounced increases in incidence occurred in Equatorial Guinea (684.19%), Angola (516.20%), Benin (453.77%), and Qatar (404.31%). The most significant declines were observed in the United States Virgin Islands, Japan, Albania, Italy, and Poland, with reductions exceeding 30% in some cases. A similar pattern was observed for ASPR, with Central, Southern, and Eastern European regions with Japan being the sole Asian nation showing declining trends, while the most substantial prevalence growth was noted in Equatorial Guinea (771.92%), Qatar (719.56%), the United Arab Emirates (546.23%), Angola (507.37%), Maldives (465.94%), Djibouti (462.16%), and Benin (445.14%). Only 13 countries or territories experienced a decrease in prevalence over the 31-year period, compared with 191 with increasing trends. Interestingly, four countries bordering Italy across the Adriatic Sea—Albania, Bosnia and Herzegovina, North Macedonia, and Serbia—exhibited paradoxically low ASPR, all below 200 per 100,000 females.

### 3.2 Joinpoint regression analysis

**Figure 3:**
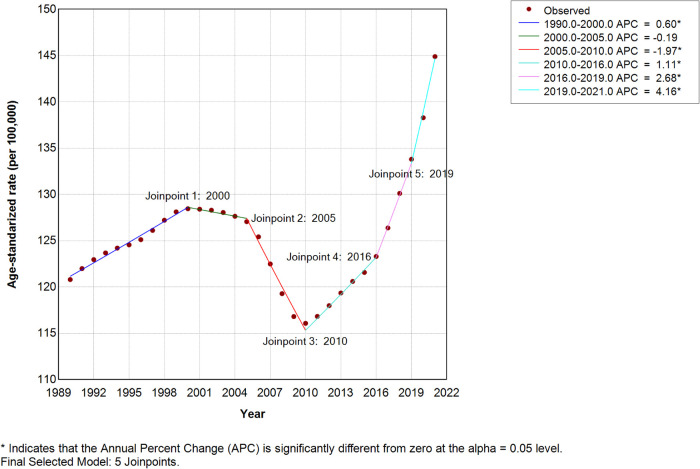
Joinpoint regression analysis of the ASIR of high SDI from 1990 to 2021.

Considering the inclusion of zero within the 95% CI of EAPC, we implemented Joinpoint regression to assess potential confounding effects on trend analyses. The average annual percentage change (AAPC) for high SDI regions showed significant increases: ASIR = 0.5743 (95% CI: 0.4689–0.6798; *P* < 0.001), ASPR = 0.5322, and ASDR = 0.5138 (Figure 3). The ASPR and ASDR of high SDI Joinpoint were presented in Supplementary Figure S1; all Joinpoint regression results consistently indicated upward trends in ASIR, ASPR, and ASDR across these locations (Supplementary Tables S3, S4).

### 3.3 Association between ASR and SDI of PCOS in different regions and nations

**Figure 4:**
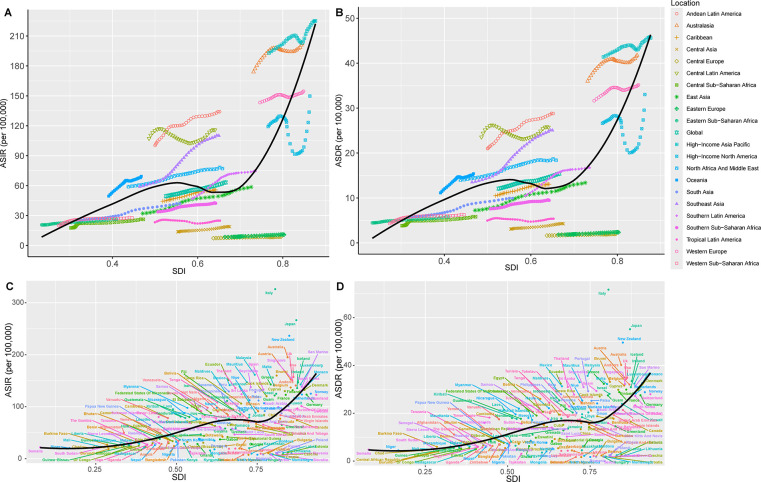
Association between age-standardized rates with SDI of PCOS across regions and nations, 1990–2021. Correlation of SDI with ASIR **(A)** and DALYs rate (ASDR) **(B)** for the different geographic regions by SDI group from 1990 to 2021. ASIR **(C)** and ASDR **(D)** for the different countries by SDI group in 2021.

The ASIR (*r*^2^ = 0.49, *P* < 0.01) and ASDR (*r*^2^ = 0.4833, *P* < 0.01) of PCOS from 1990 to 2021 performed a non-linear positive correlation with SDI, with a pattern of initial rise, followed by a decline, and then a sharp increase. The trend for change and regions mainly assembled within the SDI range of 0.4–0.7 value (Figures 4A,B). Globally, the ASIR and ASDR were below expected values in the early years between the 31 years and above in the later years with the increase in SDI. Regions such as high-income Asia Pacific, Australasia, Andean Latin America, Central Latin America, Southeast Asia, North Africa and Middle East, and Oceania had higher than expected values of ASPR, ASIR, and ASDR all the time. Other regions were mostly below expected values. Southern Latin America and the Caribbean, as the global trend, had higher than expected values in recent years. Notably, in high SDI regions, North America and the Middle East, ASIR and ASDR of PCOS were above expected values only in the first decade (1990–2021) and then declined significantly before a sharp rise after 2015 (Figure 4). Their ASPR correlation changes mirrored those of ASIR and ASDR (Supplementary Figure S2A, Supplementary Table S5).

In 2021, a positive correlation was found between SDI value with ASIR (*r*^2^ = 0.2758, *P* < 0.01) and ASDR (*r*^2^ = 0.2839, *P* < 0.01), indicating a higher incidence and burden of PCOS in higher SDI countries. However, significant variations were observed among individual countries. For example, the developed countries with high SDI, such as Canada, Norway, Switzerland, and Germany, as well as developing countries such as China, the United Arab Emirates, and Dominica, had lower ASIR, ASDR, and ASPR than expected. Conversely, the developed countries such as Italy, Japan, New Zealand, and Australia had a disease burden significantly higher than expected. Similarly, developing countries such as Mauritius, Chile, Somalia, Niger, Chad, Mali, South Sudan, and Saint Kitts and Nevis had higher than expected ASIR (Figure 4, Supplementary Figure S2B).

### 3.4 Correlation of EAPC with ASPR, ASIR, and ASDR

**Figure 5:**
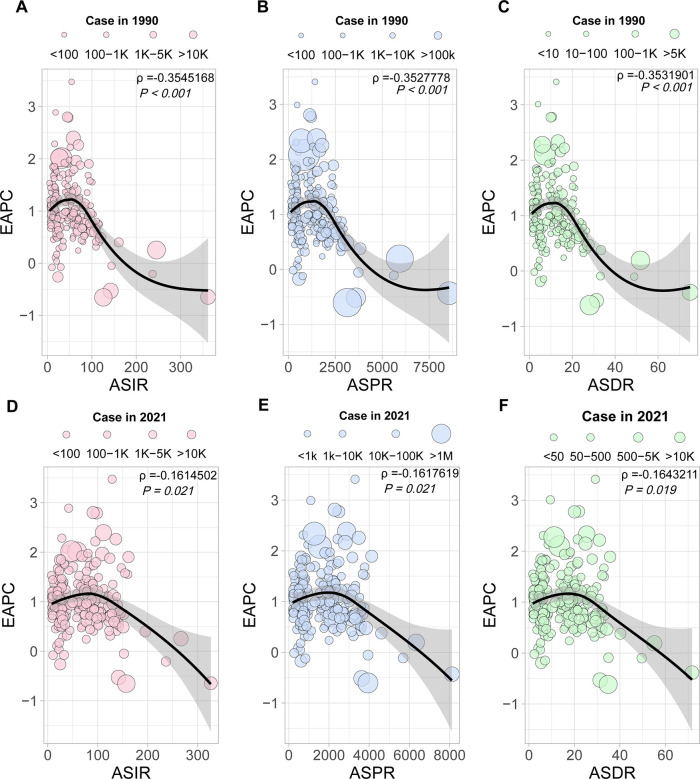
The correlation analysis of EAPC with ASIR, ASPR, and ASDR. Correlation of EAPC with ASIR (**A**), ASPR (**B**), and ASDR (**C**), the 1990 cases as a reference. Correlation of EAPC with ASIR (**D**), ASPR (**E**), and ASDR (**F**), the 2021 cases as a reference.

To analyze the association of EAPC with incidence and prevalence at the national level, a Pearson analysis method was performed for the correlation of the number and ASR of PCOS incidence, prevalence, and DALYs with its EAPC. At the national level, there is a negative correlation in EAPC with ASR of incidence, prevalence, and DALYs both in 1990 and 2021, with statistically significant differences. The Loess fitting correlation *ρ* values are presented in Figure 5 and Supplementary Table S6 (1990 ASIR, *t* = −5.3886, *P* < 0.01; 1990 ASPR, *t* = −5.3584, *P* < 0.01; 1990 ASDR, *t* = −5.3656, *P* < 0.01). Countries with high ASPR and ASIR exhibited a slower growth rate in PCOS prevalence and incidence. In regions or countries with high ASDR, its EAPC was relatively low. In 2021, when comparing the 204 countries' changes to those of 1990, a negative association was found, with the correlation weakening compared with 1990 (2021 ASIR, *t* = −2.3251, *P* = 0.021; 2021 ASPR, *t* = −2.3298, *P* = 0.021; 2021 ASDR, *t* = −2.3676, *P* = 0.019). The distribution pattern of the countries has also changed, shifting from a concentrated to a more dispersed pattern. In addition, when the country's human development index (HDI) value is 0.73, its EAPC value for incidence, prevalence, and DALY cases will begin to decrease (Supplementary Figure S3 and Supplementary Table S7). This suggests that in nations with high PCOS incidence and prevalence, as the population grows and socioeconomic development advances to a particular stage, the occurrence of PCOS will stabilize or subsequently decrease gradually.

### 3.5 Age level

To investigate the age-specific epidemiology of PCOS globally, the GBD 2021 study provided comprehensive data spanning females aged 10–54 years, stratified into nine 5-year age groups, encompassing adolescence (10–19 years), reproductive age (20–44 years), and perimenopausal stages (45–54 years). We conducted a comprehensive analysis of age-stratified incidence, prevalence, and DALYs, including both its cases and rates.

**Figure 6:**
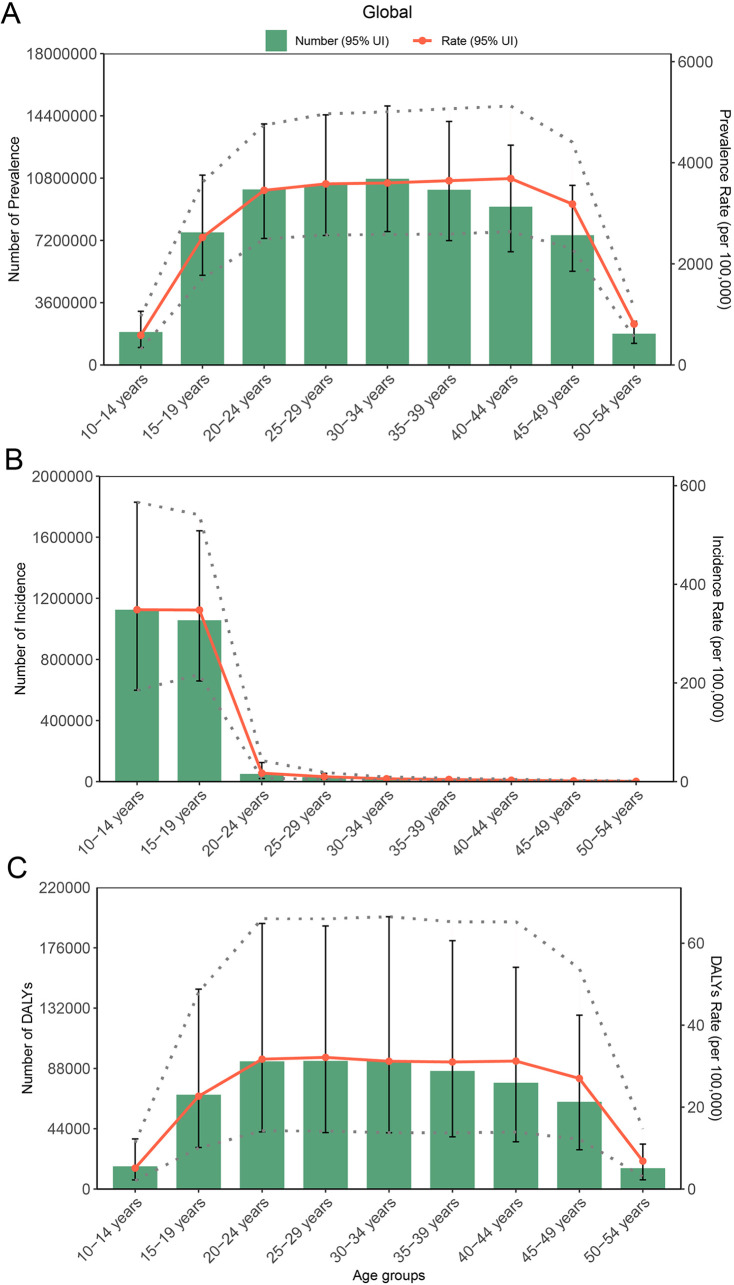
Cases and rate of incidence, prevalence, and DALYs for PCOS globally by age group, in 2021: (**A**) prevalence, (**B**) incidence, and (**C**) DALYs.

As depicted in Figure 6, in 2021, the global ASPR peaked in the 30–34-year group [3,600.5 per 100,000 females (95% CI: 2,579.8–5,007.3)] and was the lowest level in the 10–14-year group [588.5 (95% CI: 311.6–960.1)]. Absolute case counts mirrored this pattern, with the highest burden in the 30–34-year group [10,763,002.84 cases (95% CI: 7,711,792.5–14,968,296.0)] but the lowest in the 50–54-year group [1,805,283.8 cases (95% CI: 1,249,484.4–2,521,080.4)]. Incidence rates were highest in the youngest cohort (10–14 years) before progressively declining with advancing age; the cases that changed mirrored this pattern. Conversely, DALY rates reached their maximum in the 25–29-year group, while the absolute DALY cases were minimized in the 50–54-year group. These findings reveal distinct patterns between standardized rates and absolute burden metrics across the lifespan.

Furthermore, we analyzed age-stratified PCOS epidemiology across SDI quintiles, four-level health system, and geographical regions (Supplementary Figures S4–S8 and Supplementary Table S8). In 2021, the highest prevalence rates were observed in the high, high-middle, and middle SDI quintiles among the females aged 20–24-year group. In contrast, low-middle and low SDI regions peaked in older cohorts: the 35–39-year group and 30–34-year group, respectively. Notably, high-middle, middle, and low-middle SDI groups all demonstrated maximal incidence rates in the 10–14-year group and minimal rates in the 50–54-year group. Incidence case changes followed a consistent pattern. For DALY rates, a distinct pattern emerged with peak burdens in the 20–24-year group across most regions, except high-middle and low SDI quintiles, where maximum rates occurred in the 25–29-year group and minimal rates in the 10–14-year group, underscoring age-dependent variations in disease burden severity(Supplementary Table S8). Particularly, the middle SDI quintile exhibited minimal DALY rates in the 50–54-year group. We methodically evaluated age-specific incidence, prevalence, and DALY metrics (numbers and rates) across four-tiered health system capacity levels and 26 geographical subregions. Complete analytical outcomes, including stratified statistical comparisons, are also cataloged in Supplementary Table S8.

**Figure 7:**
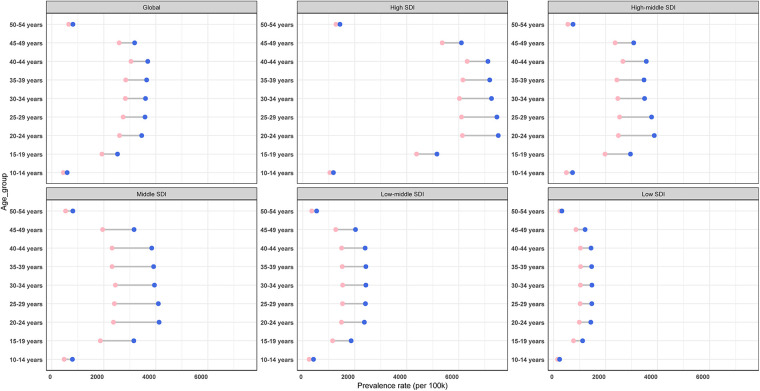
Prevalence rate for PCOS in global and different SDI regions, by age group, in 1990 (pink balls) and 2021 (purple balls).

To delineate the epidemiological transition of PCOS, we conducted comparative assessments of ASPR across global females and SDI quintiles between 1990 and 2021. Temporal analysis revealed universal ASPR increases in all SDI strata, except for the 10–14-year group and 50–54-year group, which showed relatively stable trends. The most pronounced accelerations occurred in the 20–24-year group within high, middle, and high-middle SDI regions. Low SDI regions demonstrated comparable growth magnitudes across all age groups. Notably, high SDI regions maintained substantially elevated prevalence rates among females aged 20–49 years throughout the study period, while low SDI regions persistently exhibited the lowest burdens (Figure 7).

### 3.6 Time cohort trend

**Figure 8:**
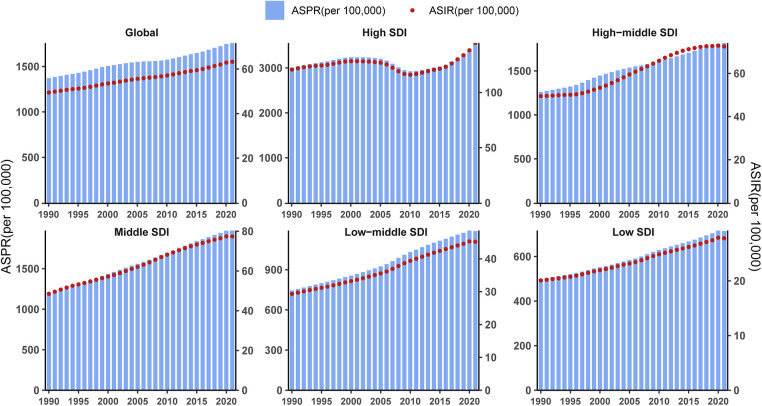
Age-standardized prevalence rate and incidence rate for PCOS in different SDI regions based on time series from 1990 to 2021.

For comparative purposes, global ASIR and ASPR values from temporal cohorts were plotted on a unified scale. From 1990 to 2021, a positive trend annually in the global ASIR and ASPR of PCOS was observed in the time cohort. And the change pattern of ASIR and ASPR is similar in all SDI quintiles. Except for high SDI, ASPR, and ASIR in the other SDI quintiles, which rose similarly to the global trend. In high SDI, PCOS ASPR and ASIR alone experienced significant fluctuations: slow growth before 2001, a decline from 2001 to 2010, and a marked rise after 2010 (Figure 8).

### 3.7 ARIMA and BAPC forecasting the global PCOS burden from 2022 to 2042

**Figure 9:**
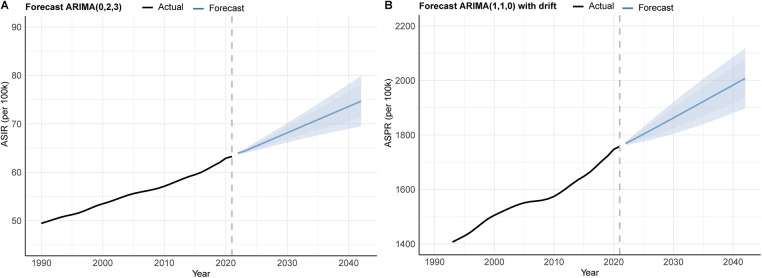
ARIMA forecast for global ASR of PCOS from 2022 to 2042: (**A**) incidence and (**B**) prevalence.

The ARIMA methodology, a widely adopted statistical framework for time series forecasting, was implemented following rigorous stationarity verification. Based on global ASIR and ASPR data spanning 1990–2021, the ASIR series was best fitted by an ARIMA(0,2,3) model (AIC = −39.38, AICc = −37.78, BIC = −33.77), while the ASPR series was modeled by ARIMA(1,1,0) with drift (AIC = 165.79, AICc = 166.68, BIC = 170.09). Both models passed the Ljung–Box test (*P* > 0.05), confirming the independence of residuals. Model outputs predict sustained increases in PCOS burden over the next two decades. By 2042, the female ASPR is projected to escalate from 1,757.8 per 100,000 population (2021 baseline) to 2,007.5 per 100,000 population, while ASIR would rise from 63.26 to 74.68 per 100,000 population (Figure 9 and Supplementary Table S9). As the trend of ARIMA, the prevalence and incidence cases of PCOS in the next 20 years will increase annually. By 2042, the global ASPR and ASIR of PCOS will be up to 3484.39 per 100,000 population and 117.41, respectively, according to estimates. These trajectories correspond to relative increases of 14.22% for ASPR and 18.05% for ASIR over the 20-year projection window, underscoring the growing global health impact of PCOS.

**Figure 10:**
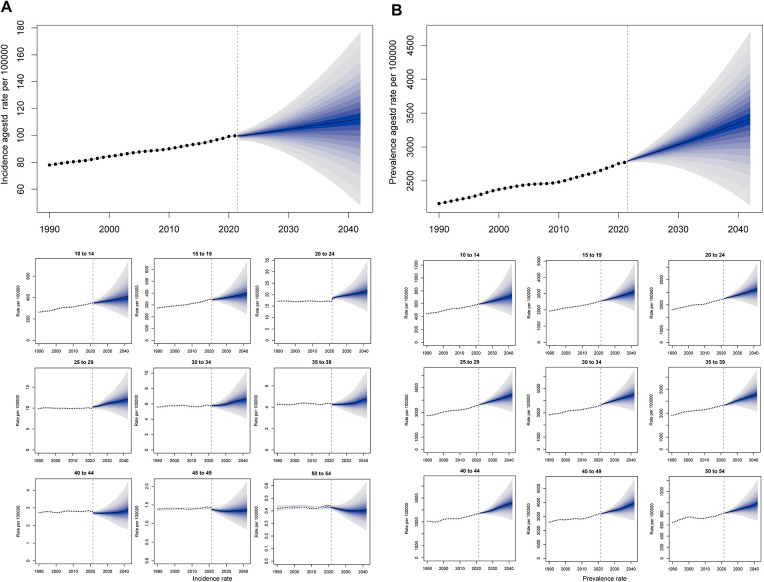
BAPC forecast for the global ASR of PCOS from 2022 to 2042. (**A**) ASIR and incidence rate of age groups from 1990 to 2042. (**B**) ASPR and prevalence rate of age groups from 1990 to 2042.

Complementarily, the BAPC model was employed to incorporate age-specific effects. The BAPC was an analysis and prediction model for time series, including age, year, and birth cohort, which relies on Bayesian. Sensitivity analysis confirmed that the results of △DIC and △CRPS were below 2, and prior specifications did not substantially affect the projected model of ASIR and ASPR fit. The results presented in Figure 10 and Supplementary Table S10. In 2024, the maximal ASPR will appear in the age group 40–44 years (4,630.44 per 100,000 population), and the maximal ASIR will show up in the age group 10–14 years (414.89).

**Figure 11:**
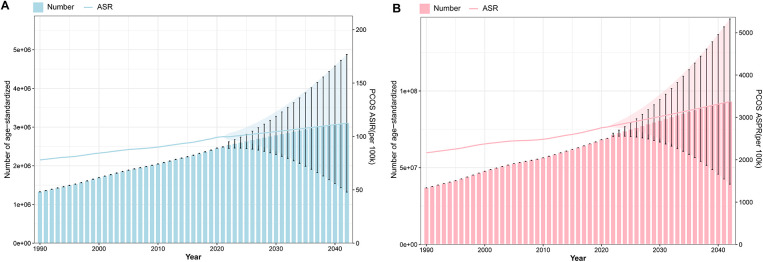
Projection of ASR and cases of PCOS global burden from 1990 to 2042. (**A**) The ASIR of BAPC with the incidence cases. (**B**) The ASPR of BAPC with the prevalence cases.

Integrated analysis of BAPC model-standardized ASIR and ASPR alongside their corresponding cases from 1990 to 2042 revealed sustained upward trends in global PCOS incidence and prevalence. The projections indicate that by 2042, incidence cases will approach 3,100,252, while prevalence cases will climb to 94,314,012. Concurrently, the ASR are anticipated to rise steadily, with ASIR expected to attain 112.35 per 100,000 population and ASPR projected to reach 3,417.76 by the target year (Figure 11 and Supplementary Table S11).

## 4 Discussion

This study provides a comprehensive report and updated assessment of the ASIR, ASPR, and ASDR for PCOS across global, regional, and national levels from 1990 to 2021. Our findings elucidate significant epidemiological dynamics of PCOS, demonstrating distinct geographical variations and age-specific patterns in disease distribution. The analysis further delineates complex interactions between healthcare accessibility, SDI, and HDI, revealing critical disparities in PCOS manifestation across different global age populations. Compared with earlier GBD 2017 and 2019, the GBD 2021 database provides more updated information on PCOS burden across time, age, sex, region, and sociodemographic groups. Moreover, beyond examining ASIR, ASPR, and ASDR by age, period, and SDI level, this study innovatively assesses the influence of health system levels on PCOS burden, an aspect previously overlooked in research (13, 18, 22, 23).

Consistent with the trends of previous research, between 1990 and 2021, the global ASIR, ASPR, and ASDR for PCOS exhibited a substantial increase trajectory (11, 16, 22, 24, 25). By 2021, the global prevalence of PCOS reached 69.47 million cases, with 2,301,505.64 incidence cases and 6,077,568.7 DALYs attributable to the condition. The EAPC analysis for PCOS burden demonstrated consistent year-on-year increases in global ASIR, ASPR, and ASDR throughout this period. By 2021, the ASIR, ASPR, and ASDR of the global population had increased to 63.26, 1,757.83, and 15.40 per 100,000 females, respectively, from 49.45, 1,372.77, and 12.08 per 100,000 in 1990. The EAPC of the three ASR metrics were calculated to 0.77 (95% CI: 0.75–0.79), 0.74 (95% CI: 0.70–0.77), and 0.72 (95% CI: 0.68–0.75), respectively.

PCOS is a polygenic genetic susceptibility disorder, and some clinical studies have verified that the mode of inheritance of PCOS may be autosomal dominant or polygenic (26). Genetic factors may play an important role in its pathogenesis, which may contribute to the escalating global burden of PCOS (27). In addition, the evolving diagnostic criteria and improved diagnostic capabilities worldwide may constitute one of the primary drivers behind the escalating PCOS burden (28). Accelerated globalization and urbanization over the past two decades have coincided with substantial global population growth, an expanding demographic base, and heightened health awareness. This increased emphasis on disease recognition has driven greater proactive healthcare-seeking behaviors, thus improving diagnostic detection rates for PCOS, which consequently amplifies its reported incidence and prevalence (29). In the contemporary era of continuous development, persisting disparities in healthcare, education, and social development across nations and regions continue to shape the burden of PCOS. Factors such as the adequacy of healthcare infrastructure, women's access to education and health information, and their living environments and socioenvironmental conditions may significantly influence these disparities (18). A study in the United States highlighted the need to address barriers faced by gynecologists and primary care providers in implementing PCOS diagnosis and management guidelines, underscoring the importance of adopting targeted multidisciplinary care models (30).

Considering that enhancing the health systems is one of the eleven global priorities emphasized by the GBD database study organization, we conducted further analysis for the different levels of SDI and health systems. In subsequent analyses, we observed that high SDI and advanced health systems exhibited markedly elevated levels of ASPR, ASIR, and ASDR compared with other regions. Notably, these metrics demonstrated an initial decline followed by a subsequent increase over time, a temporal pattern distinct from trends observed in regions with different SDI classifications. These disparities may stem from differential diagnostic capabilities in healthcare systems and sociocultural influences on healthcare-seeking behaviors (31). High SDI, typically associated with advanced socioeconomic development, demonstrates greater national prioritization of PCOS screening, treatment, and management. Concurrently, the comprehensive healthcare infrastructure and sophisticated medical capabilities in regions with advanced health systems have led to elevated PCOS detection rates.

Furthermore, PCOS patients in these regions benefit from more comprehensive long-term management strategies encompassing lifestyle interventions, pharmacotherapy, and surveillance for long-term complications. The post-2003 gradual standardization of diagnostic criteria has reduced false-positive rates arising from inconsistent application (e.g., overdiagnosis in adolescent populations), which may explain the observed burden reduction in high SDI and advanced health systems during specific time periods (32). After 2010, the accelerated PCOS burden escalation in high SDI and advanced health systems was likely driven by demographic shifts, heightened health awareness, refined diagnostic methodologies with expanded primary care implementation, increasing precision of ultrasound techniques, and the growing prominence of obesity linked to high-calorie dietary patterns (33). In Australia, the estimated annual healthcare costs related to PCOS exceed 800 million AUD. The National Women's Health Strategy 2020–2030 outlines a national approach to improving health outcomes for all women and girls, identifying PCOS as a priority in reproductive health. In the United States, the Senate has formally recognized the seriousness of PCOS and designated September as PCOS Awareness Month, significantly elevating awareness among both policymakers and patients (34).

In Asia, India, one of the largest contributors to global population growth, has also established tailored clinical guidelines for PCOS. These guidelines leverage collaborative multidisciplinary approaches to support high-quality diagnosis and management, while providing an evidence-based framework to standardize care across diverse clinical settings (35, 36).In contrast, low SDI and limited health system capacity face critical challenges: insufficient medical technology, shortages of specialized healthcare personnel, and constrained accessibility to PCOS-related care (37). These systemic limitations result in inadequate screening coverage, restricted diagnostic and therapeutic capacities, and high rates of underdiagnosis with low detection rates, leaving numerous potential cases undiagnosed. Compounding these issues, populations in these regions exhibit lower awareness of PCOS symptoms and frequently delay medical consultations due to financial constraints (38, 39). Moreover, therapeutic management for diagnosed cases often proves suboptimal, with most diagnoses occurring only when quality-of-life-impairing symptoms manifest (40). The long-term health consequences of PCOS in these settings often remain unaddressed, leading to substantial underestimation of the true PCOS burden. And the correlated result of SDI with PCOS's ASR showed that a higher SDI value presented a high disease burden. Even though the PCOS prevalence rate is low in the regions of middle SDI value, it also dramatically changes when the SDI value is up to 0.73. The converging patterns of ASR for prevalence, incidence, and DALYs in their associations with the SDI suggest remarkable consistency in PCOS epidemiology across geographic strata. This parallelism implies an absence of abrupt epidemiological transitions in PCOS distribution, with disease burden fluctuations predominantly aligning with established socioeconomic development trajectories rather than exhibiting region-specific anomalies. However, there is growing recognition of the importance of PCOS treatment in low and low-middle SDI countries. Nations such as Ethiopia and Bhutan, for instance, are implementing effective strategies to address gender-based health inequities (41, 42).

Differences and changes in the heterogeneity in PCOS burden exist across geographic regions and nations. In 2021, high-income Asia Pacific, the region with the heaviest PCOS burden, demonstrated over 20-fold differences in ASIR and ASPR compared with Central Europe, which exhibited the lightest disease burden. Japan, New Zealand, Malaysia, and Singapore contributed more than other countries in the process. This phenomenon may arise from differences in the distribution of PCOS susceptibility genes across geographic locations and ethnic populations (17). Variations in living environments, lifestyle patterns, socioeconomic development levels, and accessibility of healthcare resources in different regions or nations may further contribute to the heterogeneous burden of PCOS (43). This observation underscores the demonstrable influence of diagnostic protocol evolution and implementation scope on disease burden metrics. Notably, Japan has developed revised diagnostic and management guidelines tailored to its specific pathological profile of PCOS, distinct from those in Europe and the United States. Similarly, Italy established nationally adapted diagnostic and management guidelines, consensus statements, and recommendations. These efforts have enhanced the clinical management of infertility in women with PCOS, a response correlated with the country's notably high ASIR and ASPR (44, 45). Notwithstanding elevated caseload baselines, Italy and Japan exhibited downward trajectories in PCOS, while Germany, Austria, Greece, and Romania demonstrated <15% fluctuations in ASPR and ASIR. This epidemiological stabilization pattern suggests successful modulation of PCOS progression, potentially attributable to impactful care coordination and advanced healthcare delivery systems in these developed nations. In addition, regional discrepancies in data quality, sampling biases, and methodological approaches in epidemiological studies may introduce variability in PCOS burden estimations across different jurisdictions (46).

At the age and time series level, our results show no significant difference from the previous studies. Notably, the global incidence of PCOS patients in 2021 is highest in the age group of 10–14 years, with a decline in the number of incidence cases in the higher age groups. This peak likely correlates with pubertal hormonal fluctuations, as adolescence involves physiological transitions marked by menstrual irregularities, acne, and rapid endocrine changes, features overlapping with PCOS diagnostic criteria, potentially inflating detection rates during this developmental phase. High body mass index (BMI) emerges as a key risk factor contributing to the elevated incidence of PCOS in adolescent populations (25). With advancing age, stabilization of endocrine regulation may contribute to reduced incidence of cases. However, the substantial diagnostic ambiguity when applying existing criteria to adolescent populations underscores the need for age-specific diagnostic frameworks to distinguish between normal pubertal changes and pathological manifestations (47, 48). Consistent with 1990 observations, the highest PCOS prevalence globally in 2021 was observed in the 40–44-year group, potentially reflecting cumulative exposure to risk factors over time. Age-related exacerbation of PCOS-associated metabolic disturbances (e.g., insulin resistance, obesity) and endocrine dysregulation likely drives this prevalence escalation. Notably, peak DALYs occurred among women aged 25–29 years, a critical window coinciding with prime childbearing years. The elevated disease burden in this cohort stems from PCOS-related reproductive complications (such as infertility, miscarriage risks) and accelerated development of metabolic comorbidities, such as type 2 diabetes, cardiovascular risks, collectively amplifying health loss metrics during this life stage.

Our correlation analyses revealed negative associations between ASPR, ASIR, ASDR, and EAPC or HDI. This suggests that while PCOS continues to pose substantial health burdens, its growth trajectory shows signs of deceleration in regions with established disease management strategies. The influence of PCOS on population growth slows down. These nations or areas may benefit from technological advancements in healthcare and sustained long-term interventions that moderate the escalation rate of PCOS-related health impacts. Using ARIMA and BAPC models, we projected global PCOS burden trends over the next two decades. Forecasts indicate persistent increases in ASPR and ASIR, with anticipated rises of 18% in ASIR and 14% in ASPR by 2042, peaking in 10–14 year age groups. This progression likely stems from multiple interacting factors: demographic and lifestyle transitions, enhanced diagnostic capacity coupled with improved disease recognition, environmental endocrine disruptor exposure (e.g., bisphenol A), and psychosocial stressors.

These findings highlight PCOS burden escalation as an emerging global public health priority requiring urgent policy attention. Strategic responses should prioritize optimized healthcare resource allocation, early screening protocols, multidisciplinary care frameworks, and robust public health education initiatives and focus on the adolescent females' reproductive development issues to mitigate population-level impacts. Despite its substantial disease burden, PCOS remains disproportionately underfunded relative to other reproductive disorders such as endometriosis. This research investment gap necessitates coordinated governmental action to develop multisectoral health policies that empower affected women and families, thereby mitigating PCOS progression, improving gynecological health outcomes, and advancing reproductive health equity (49). Integrating endocrinology services into primary healthcare through task-shifting strategies could improve early detection rates in resource-limited settings, while public–private partnerships may accelerate development of affordable diagnostic tools tailored for diverse phenotypic presentations (50–53).

This investigation constitutes the first multinational-level synthesis of PCOS epidemiology utilizing GBD 2021 data, comprehensively analyzing and comparing incidence, prevalence, and DALY metrics across 204 nations and 21 regions. The standardized epidemiological analysis results provide critical benchmarks for global health authorities, enabling evidence-based cross-jurisdictional comparisons to optimize strategic resource prioritization and advance equity-focused healthcare policies for PCOS management worldwide.

## 5 Limitations

Heterogeneity in diagnostic criteria and clinical manifestations across regions/nations introduces potential data bias in PCOS burden assessments. Unlike other conditions, PCOS often evades timely detection, with many cases diagnosed years after symptom onset, perpetuating underestimation of its true prevalence and incidence. Despite the sophisticated methods employed by the GBD study to collect data, limitations remain. Systematic underdiagnosis and underreporting of PCOS, particularly in regions with limited access to healthcare, are persistent challenges that may lead to an underestimation of the true burden. In addition, inherent limitations in GBD data collection methodologies, such as incomplete medical record systems in certain countries, contribute to underreporting and diagnostic gaps. Our analysis reveals that the GBD database currently captures PCOS data exclusively for females aged 10–54 years. Given ongoing sociodemographic shifts and physiological evolution, PCOS demographics are expanding beyond this range. However, the accuracy of our estimates is ultimately contingent on the quality and quantity of the underlying GBD2021 source data. First, to address these challenges, future efforts should prioritize harmonizing diagnostic standards while accounting for ethnic/geographic variations in clinical presentation. Subsequent PCOS diagnostic guidelines and consensus statements, accepted by the GBD data report standard, should incorporate a broader spectrum of phenotypes and age groups to improve diagnostic inclusivity and reduce underdetection and misdiagnosis. Second, strengthening data infrastructure, integrating regional epidemiological insights, and establishing region-specific diagnostic protocols are critical. Third, broadening data collection to broader age groups and implementing longitudinal follow-ups to advance understanding of PCOS epidemiology and disease trajectory across the female life course. Targeted population studies and multicenter validation of risk factors will further reduce calibration biases, enhancing the accuracy and reliability of global PCOS burden evaluations.

## 6 Conclusion

This study confirms a 28% rise in global PCOS burden since 1990, revealing critical disparities: lower SDI regions exhibit accelerating prevalence despite limited healthcare access, while advanced SDI and health systems sustain a high prevalence rate and low incidence rate change. Peak incidence occurs in adolescents (10–19 years), with reproductive-age women (20–35 years) bearing the highest caseload, which demands urgent fertility protection policies. The key imperatives include enhancing screening access in underserved populations and implementing precision prevention frameworks accounting for regional epidemiology and age-specific risks. Critical research gaps remain in developing cost-efficient interventions, real-time monitoring systems, and context-adapted strategies for resource-constrained settings.

## Data Availability

The original contributions presented in the study are included in the article/Supplementary Material, further inquiries can be directed to the corresponding author.
