# Supplementary material for: Global trends and health system impact on polycystic ovary syndrome: a comprehensive analysis of age-stratified females from 1990 to 2021
Source: Front Reprod Health. 2025 Oct 23;7:1642369. doi: 10.3389/frph.2025.1642369 (PMC12588920; doi:10.3389/frph.2025.1642369)
Supplement: Supplementary file 1 [file Datasheet1.zip › Supplemental_data/Supplementary_Material_Figures_20250606.docx]

Supplementary Material

# Supplementary Figures and Tables

## Supplementary Figures


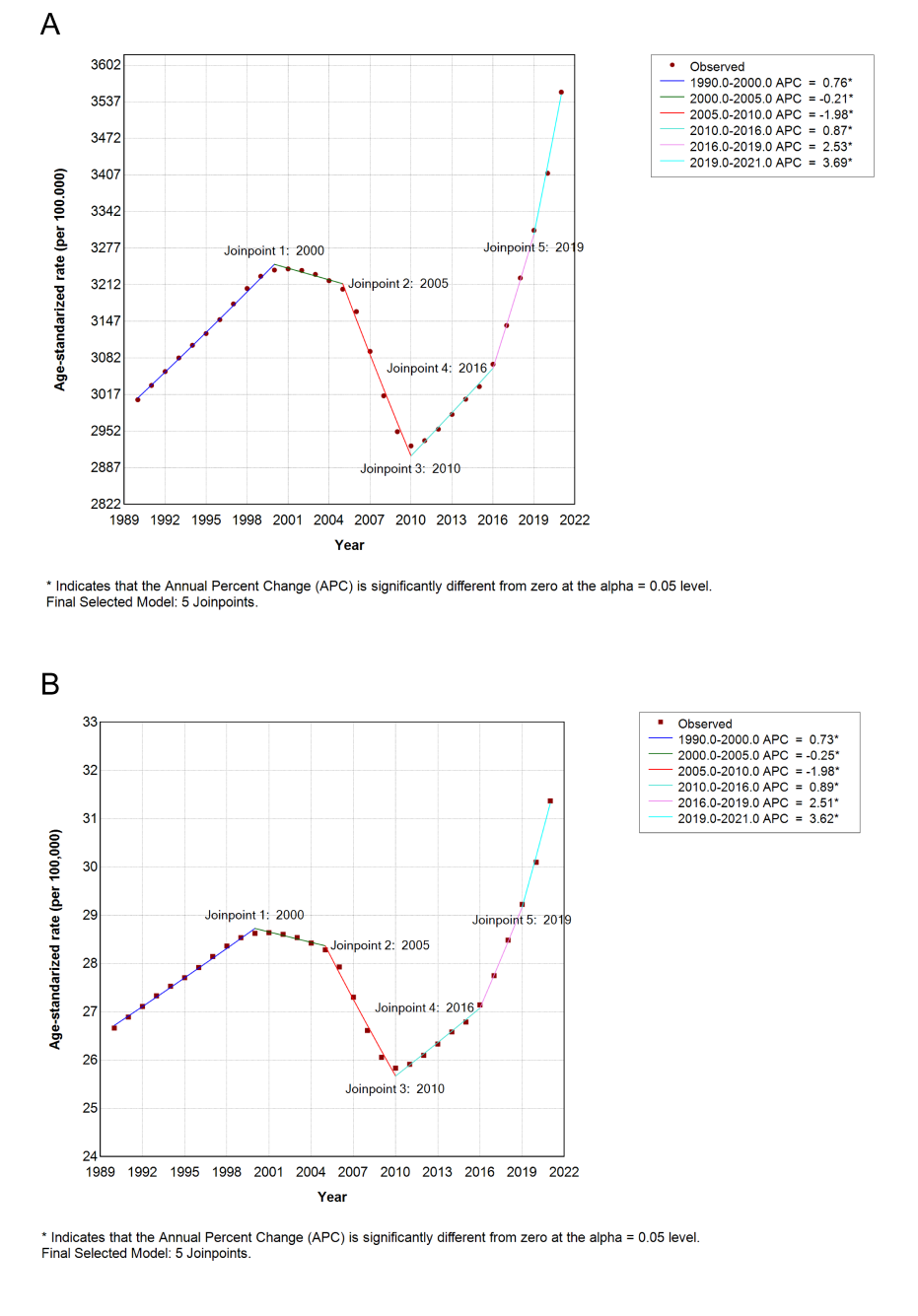


**Figure S1.** Joinpoint regression analysis of High SDI from 1990 to 2021. (A) ASPR;(B) ASDR.


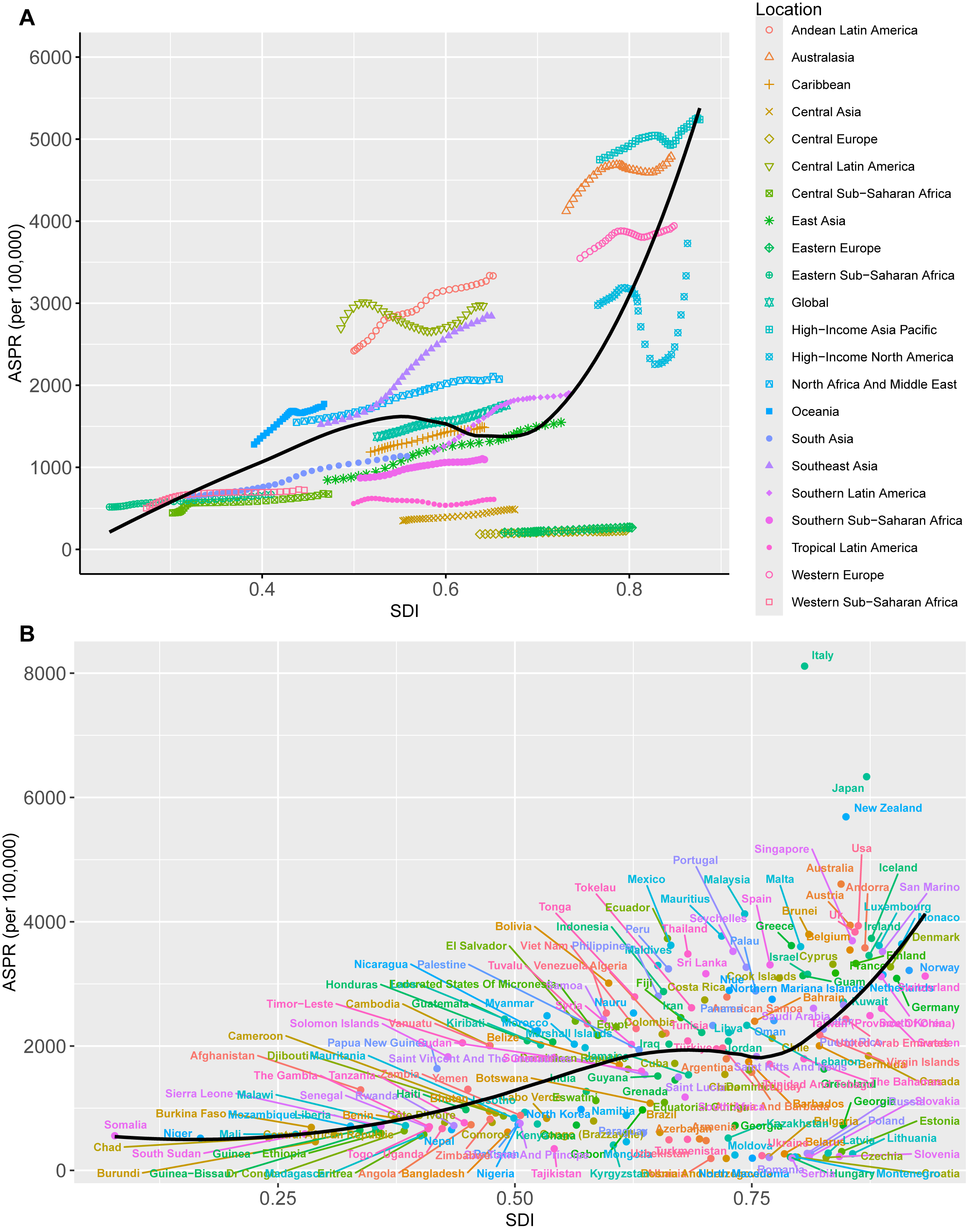


**Figure S2.**The correlation of SDI with Age-standardized prevalence rate. (A) for the different geographic regions by SDI group from 1990 to 2021; (B) for the different countries by SDI group in 2021.


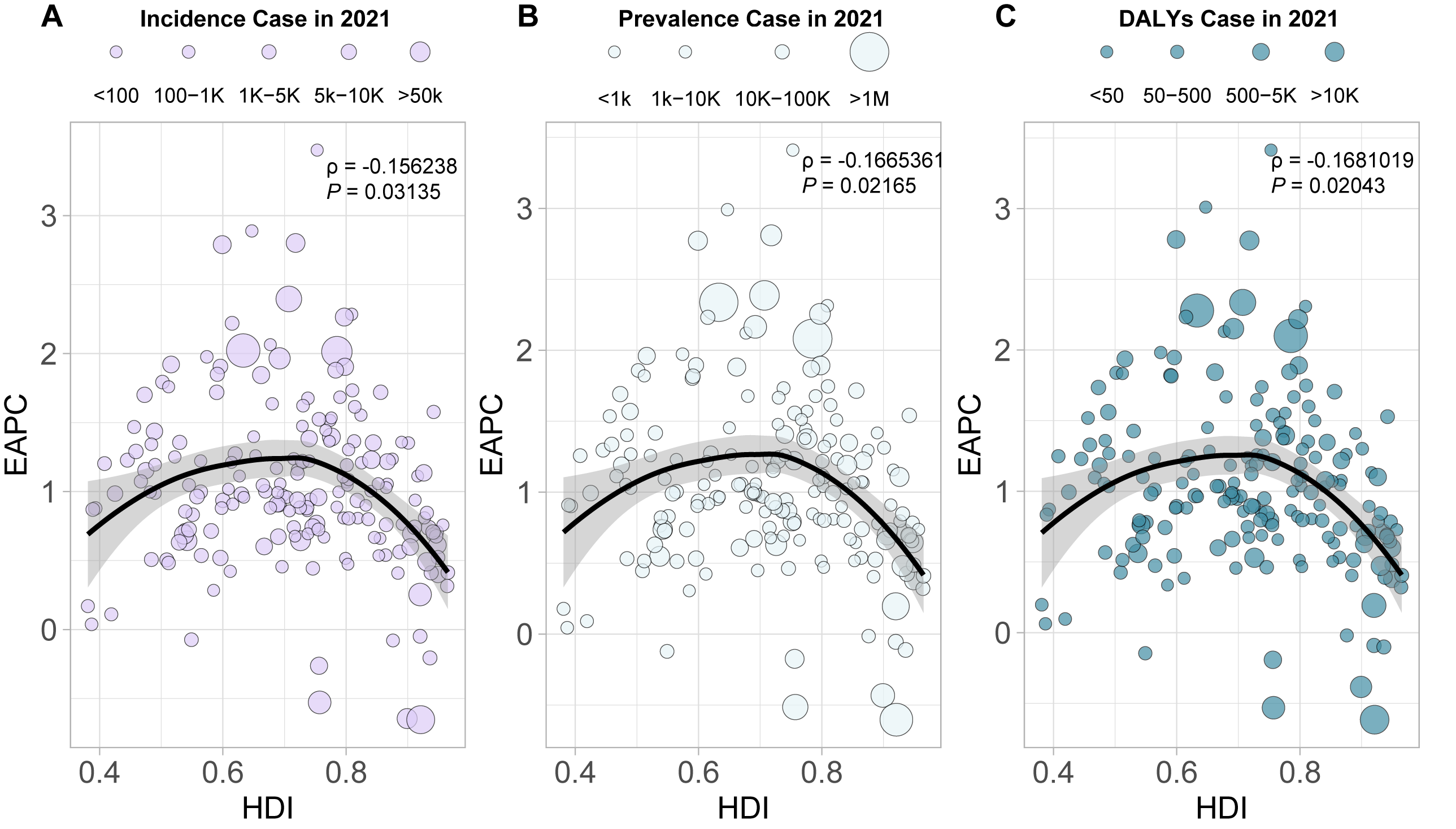


**Figure S3.** The correlation analysis of EAPC with HDI of countries. (A) incidence; (B)prevalence ;(C)DALYs cases.


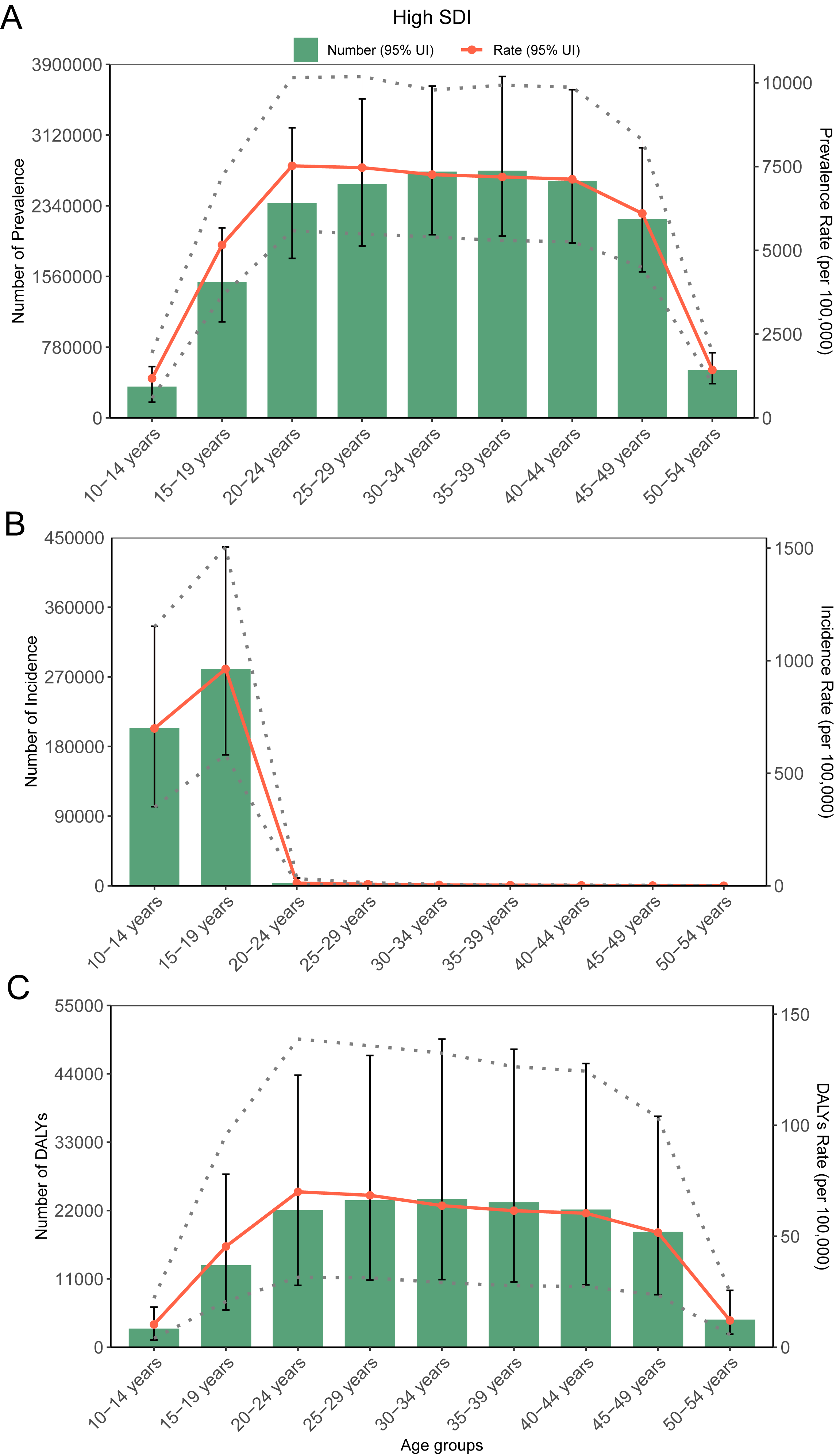


**Figure S4.** Comparison of High SDI cases with rates by age groups in 2021.(A) Prevalence; (B) Incidence; (C) DALYs.


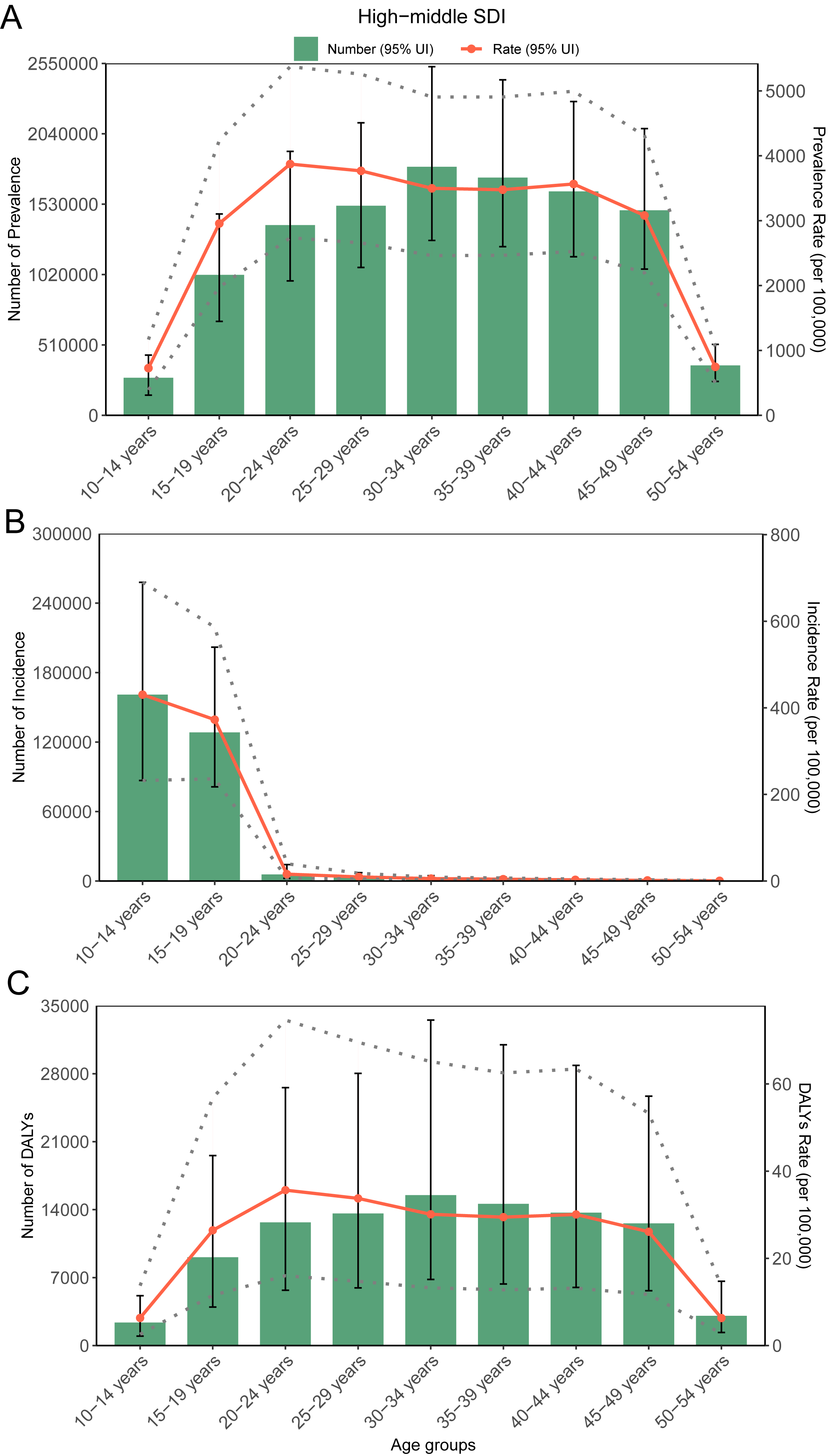


**Figure S5.** Comparison of High-middle SDI cases with rates by age groups in 2021.(A) Prevalence; (B) Incidence; (C) DALYs.


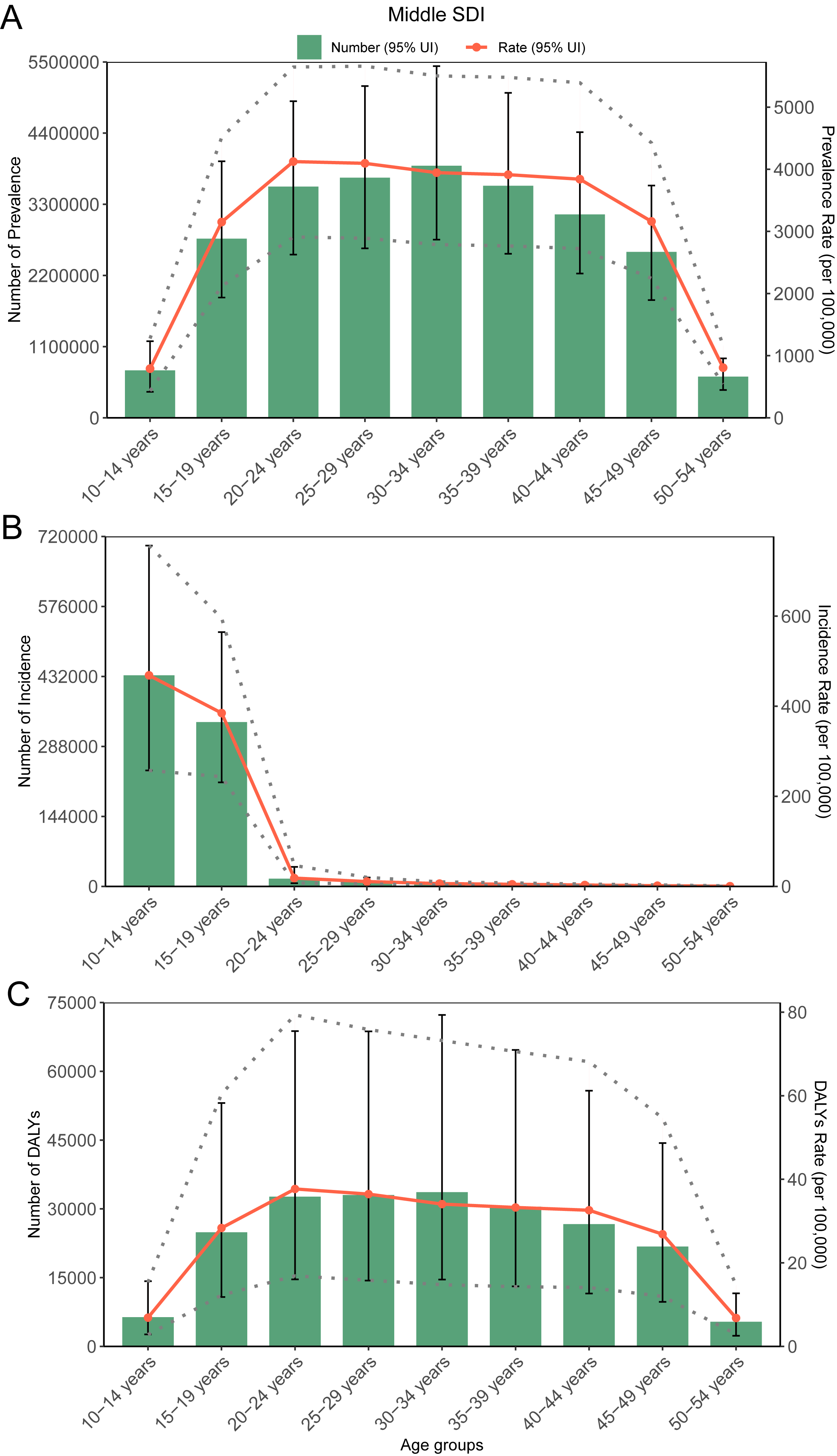


**Figure S6.** Comparison of Middle SDI cases with rates by age groups in 2021.(A) Prevalence; (B) Incidence; (C) DALYs.


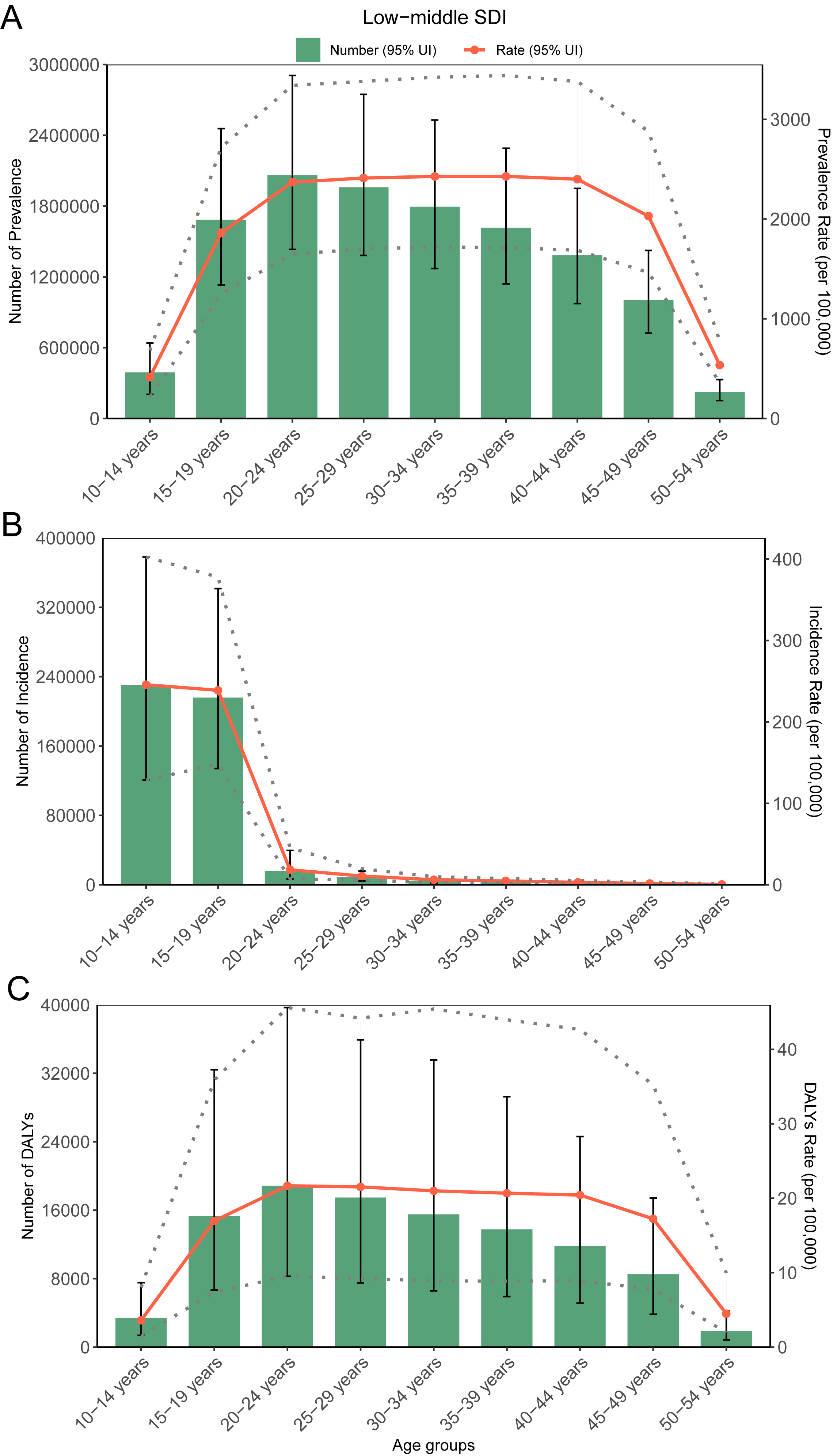


**Figure S7.** Comparison of Low-middle SDI cases with rates by age groups in 2021.(A) Prevalence; (B) Incidence; (C) DALYs.


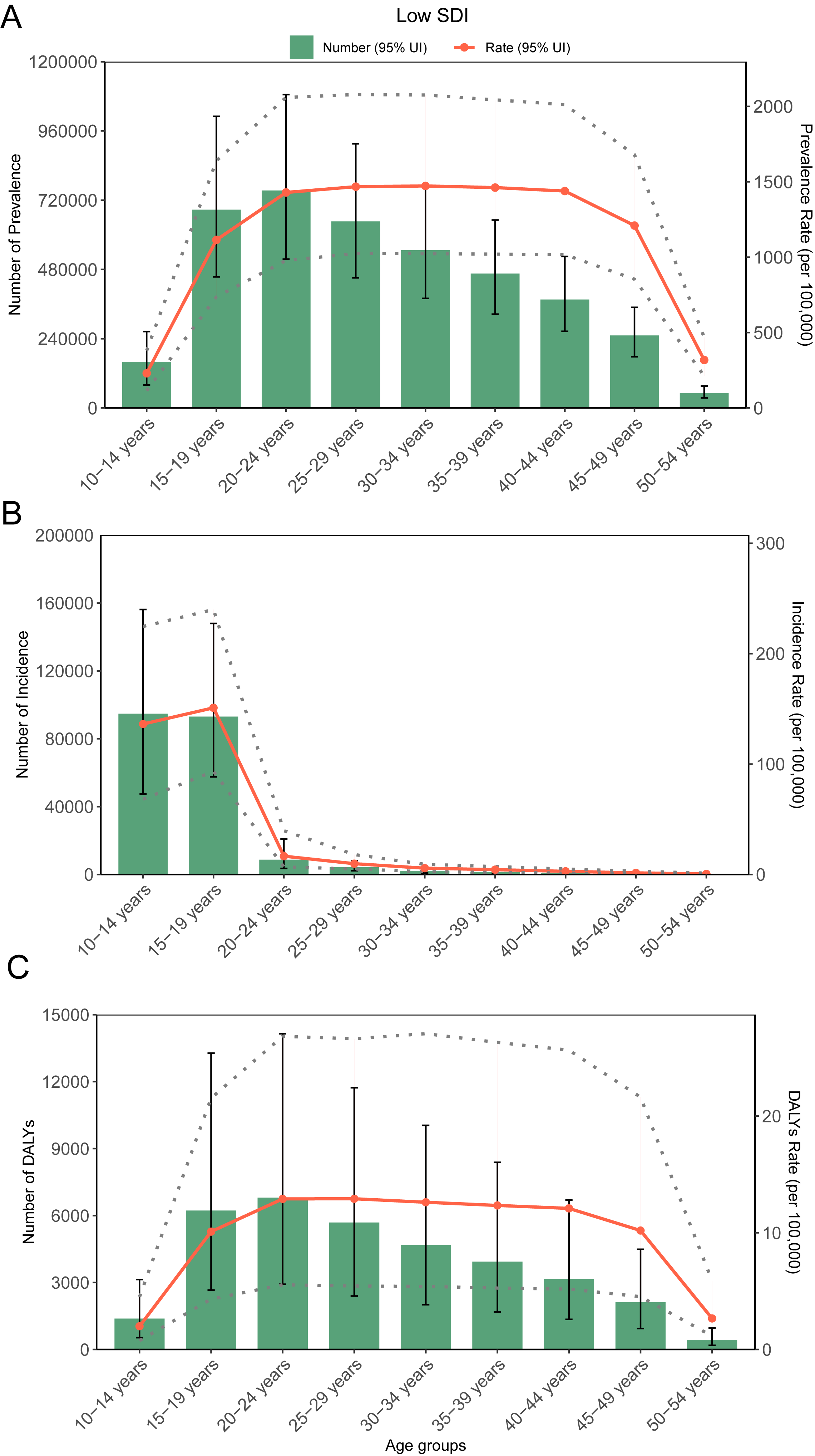


**Figure S8.** Comparison of Low SDI cases with rates by age groups in 2021.(A) Prevalence; (B) Incidence; (C) DALYs.

## Supplementary Tables

## **Table S1**. Global and regional trends in PCOS burden: prevalence, disability-adjusted life years and EAPC from 1990 to 2021.

**Table S2**. The Incidence and prevalence changes rate of PCOS in nations

**Table S3**. Joinpoint regression analysis of AAPC for ASIR, ASPR and ASDR from 1990 to 2021 in SDI, regions and countries with no significant EAPC trends.

**Table S4.** Joinpoint regression analysis results of APC for ASIR, ASPR and ASDR from 1990 to 2021 in SDI, regions and countries with no significant EAPC trends.

**Table S5**. Analysis of ASIR,ASPR and ASDR with SDI in region groups from 1990 to 2021.

**Table S6**. Relation of ASR with SDI for 204 countries in 2021.

**Table S7**. Correlation between the number, HDI and EAPC of PCOS incidence, prevalence and DALYs in 2021 among 190 countries.

**Table S8**. Related analysis of Incidence, Prevalence and DALYs' Number with rate for Age groups in different regions.

**Table S9**. ARIMA forecast of ASIR and ASPR for the global PCOS.

**Table S10.** BAPC forecast of PCOS global case and incidence-prevalence case from 2022 to 2042.

**Table S11**. Relation of PCOS Global ASR and case from 1990 to 2042.
