# Supplementary material for: Global trends and health system impact on polycystic ovary syndrome: a comprehensive analysis of age-stratified females from 1990 to 2021
Source: Front Reprod Health. 2025 Oct 23;7:1642369. doi: 10.3389/frph.2025.1642369 (PMC12588920; doi:10.3389/frph.2025.1642369)
Supplement: Supplementary file 1 [file Datasheet1.zip › Supplemental_data/Table S1. Global and regional trends in PCOS burden prevalence, disability-adjusted life years and EAPC from 1990 to 2021.docx]

Table S1.Global and regional trends in PCOS burden: prevalence, disability-adjusted life years and EAPC from 1990 to 2021.

| **Location** | **Number_1990** | **ASR_1990** | **Number_2021** | **ASR_2021** | **EAPC_CI** |
| --- | --- | --- | --- | --- | --- |
| **Prevalence** |  |  |  |  |  |
| Global | 36651157.24 ( 26227943.17 - 50603929.78 ) | 1372.77 ( 984.64 - 1891.60 ) | 69473252.37 ( 49531420.00 - 95724479.23 ) | 1757.83 ( 1253.36 - 2421.26 ) | 0.74(0.70-0.77) |
| SDI |  |  |  |  |  |
| High SDI | 13783058.46 ( 10021809.44 - 19223038.27 ) | 3007.94 ( 2186.43 - 4172.47 ) | 17573919.77 ( 12981150.82 - 23876520.56 ) | 3554.29 ( 2624.21 - 4816.07 ) | 0.09(-0.08-0.25) |
| High-middle SDI | 7047030.00 ( 4981606.26 - 9727416.64 ) | 1262.73 ( 892.37 - 1745.92 ) | 11180894.89 ( 7874794.87 - 15590881.41 ) | 1817.62 ( 1277.35 - 2529.47 ) | 1.22(1.18-1.26) |
| Middle SDI | 10580143.20 ( 7410905.26 - 14674450.81 ) | 1176.94 ( 825.22 - 1630.44 ) | 24613369.91 ( 17452531.61 - 34074253.7 ) | 1971.07 ( 1395.79 - 2724.23 ) | 1.73(1.69-1.77) |
| Low-middle SDI | 4094311.31 ( 2868780.90 - 5765430.73 ) | 745.13 ( 526.92 - 1040.82 ) | 12118405.37 ( 8434180.98 - 17027257.26 ) | 1188.74 ( 828.69 - 1668.94 ) | 1.64(1.59-1.68) |
| Low SDI | 1121930.97 ( 784807.02 - 1611458.42 ) | 500.06 ( 353.56 - 711.57 ) | 3938251.29 ( 2745129.91 - 5589751.24 ) | 714.31 ( 504.52 - 1007.40 ) | 1.23(1.20-1.26) |
| Health system |  |  |  |  |  |
| Advanced Health System | 17349361.39 ( 12584994.37 - 23781916.80 ) | 2571.04 ( 1863.55 - 3515.33 ) | 21154221.76 ( 15573659.34 - 28771671.29 ) | 3046.95 ( 2251.20 - 4160.34 ) | 0.18(0.04-0.32) |
| Basic Health System | 14066187.71 ( 9900136.11 - 19602002.01 ) | 1153.21 ( 812.45 - 1604.79 ) | 30516465.64 ( 21616756.11 - 42543674.16 ) | 1962.56 ( 1384.74 - 2730.96 ) | 1.76(1.69-1.83) |
| Limited Health System | 4925706.22 ( 3482413.50 - 6925214.68 ) | 671.39 ( 480.34 - 938.62 ) | 16662344.43 ( 11625899.06 - 23359835.47 ) | 1097.89 ( 767.23 - 1534.47 ) | 1.79(1.72-1.86) |
| Minimal Health System | 285218.60 ( 199008.70 - 413707.90 ) | 467.19 ( 328.23 - 670.70 ) | 1091809.39 ( 756623.34 - 1554184.47 ) | 684.85 ( 477.11 - 969.41 ) | 1.22(1.15-1.30) |
| Regions |  |  |  |  |  |
| Andean Latin America | 466365.78 ( 322493.99 - 653112.67 ) | 2419.72 ( 1673.59 - 3379.44 ) | 1172864.51 ( 808337.39 - 1649165.85 ) | 3333.66 ( 2301.61 - 4690.91 ) | 1.08(1.00-1.15) |
| Australasia | 444239.31 ( 326487.36 - 585953.23 ) | 4122.50 ( 3027.98 - 5452.98 ) | 701617.99 ( 500341.02 - 974912.97 ) | 4786.95 ( 3430.23 - 6607.27 ) | 0.27(0.18-0.36) |
| Caribbean | 222432.35 ( 149139.10 - 319229.69 ) | 1186.23 ( 799.13 - 1702.73 ) | 360981.83 ( 245137.34 - 520033.49 ) | 1485.97 ( 1008.31 - 2136.92 ) | 0.77(0.71-0.83) |
| Central Asia | 118817.93 ( 79820.20 - 174949.99 ) | 348.50 ( 234.84 - 511.40 ) | 237958.89 ( 163368.39 - 333464.66 ) | 483.56 ( 330.71 - 678.72 ) | 1.18(1.11-1.25) |
| Central Europe | 116151.96 ( 76720.98 - 173921.49 ) | 185.82 ( 122.77 - 278.52 ) | 119149.23 ( 82050.44 - 168514.00 ) | 228.93 ( 157.78 - 326.20 ) | 0.64(0.58-0.69) |
| Central Latin America | 2295521.99 ( 1578370.65 - 3199568.77 ) | 2695.62 ( 1856.93 - 3736.43 ) | 4072957.18 ( 2852240.56 - 5662452.40 ) | 2967.64 ( 2076.22 - 4124.04 ) | -0.08(-0.24-0.08) |
| Central Sub-Saharan Africa | 110521.94 ( 76168.15 - 160819.70 ) | 445.38 ( 309.50 - 646.83 ) | 444120.79 ( 305191.60 - 640542.02 ) | 673.86 ( 465.36 - 968.69 ) | 1.28(1.14-1.43) |
| East Asia | 5607180.87 ( 3957250.24 - 7863221.6 ) | 845.57 ( 600.94 - 1185.47 ) | 10490358.54 ( 7423407.50 - 14808757.10 ) | 1548.43 ( 1085.52 - 2170.68 ) | 2.04(1.89-2.19) |
| Eastern Europe | 237422.15 ( 160523.33 - 339244.51 ) | 206.51 ( 139.23 - 298.06 ) | 265678.69 ( 185374.43 - 381762.99 ) | 264.99 ( 182.67 - 381.43 ) | 0.98(0.93-1.03) |
| Eastern Sub-Saharan Africa | 448071.03 ( 310256.93 - 646236.16 ) | 516.67 ( 364.08 - 741.26 ) | 1438942.37 ( 1001296.83 - 2063339.49 ) | 669.17 ( 472.35 - 949.85 ) | 0.86(0.83-0.89) |
| High-income Asia Pacific | 4402355.40 ( 3172199.05 - 6106156.59 ) | 4750.21 ( 3430.32 - 6586.66 ) | 4104982.68 ( 2922772.61 - 5775182.08 ) | 5237.62 ( 3779.21 - 7307.15 ) | 0.25(0.20-0.30) |
| High-income North America | 4469583.70 ( 3157929.56 - 6274672.88 ) | 2975.32 ( 2098.69 - 4185.41 ) | 6362238.32 ( 4742961.86 - 8324162.21 ) | 3729.48 ( 2777.89 - 4876.97 ) | -0.52(-1--0.04) |
| North Africa and Middle East | 2463301.08 ( 1707181.32 - 3501813.36 ) | 1548.77 ( 1073.96 - 2200.34 ) | 6673431.49 ( 4672056.28 - 9434543.48 ) | 2075.28 ( 1453.41 - 2932.69 ) | 1.09(1.04-1.13) |
| Oceania | 40226.69 ( 27505.04 - 56891.57 ) | 1278.85 ( 881.22 - 1798.41 ) | 124484.33 ( 86570.21 - 177516.43 ) | 1772.65 ( 1232.67 - 2531.47 ) | 0.82(0.65-0.98) |
| South Asia | 3294301.20 ( 2344772.01 - 4593824.79 ) | 642.80 ( 462.57 - 893.35 ) | 11291117.08 ( 7950085.92 - 15832639.77 ) | 1135.87 ( 799.70 - 1591.05 ) | 2.13(2.05-2.22) |
| Southeast Asia | 3682948.82 ( 2589198.76 - 5201509.94 ) | 1522.31 ( 1075.34 - 2154.40 ) | 10520027.69 ( 7378813.87 - 14809823.50 ) | 2842.65 ( 1993.15 - 3997.51 ) | 2.31(2.21-2.41) |
| Southern Latin America | 296321.47 ( 204840.66 - 431020.07 ) | 1185.87 ( 822.02 - 1727.03 ) | 667604.83 ( 469021.54 - 956761.41 ) | 1892.50 ( 1326.86 - 2707.18 ) | 1.46(1.25-1.68) |
| Southern Sub-Saharan Africa | 232808.63 ( 160575.02 - 334631.38 ) | 870.65 ( 601.45 - 1246.58 ) | 480389.59 ( 328945.22 - 679589.28 ) | 1094.76 ( 749.33 - 1548.05 ) | 0.79(0.71-0.87) |
| Tropical Latin America | 448844.15 ( 304409.01 - 647659.16 ) | 558.68 ( 379.28 - 801.65 ) | 746471.68 ( 514676.97 - 1057462.85 ) | 610.00 ( 419.25 - 869.52 ) | -0.14(-0.3-0.02) |
| Western Europe | 6815142.46 ( 4796290.79 - 9455180.18 ) | 3547.26 ( 2498.52 - 4925.03 ) | 7455929.27 ( 5232259.44 - 10460556.82 ) | 3942.92 ( 2761.02 - 5529.65 ) | 0.21(0.14-0.27) |
| Western Sub-Saharan Africa | 438598.33 ( 307139.67 - 633877.78 ) | 501.95 ( 354.54 - 717.07 ) | 1741945.38 ( 1209889.21 - 2493010.70 ) | 722.68 ( 508.32 - 1026.25 ) | 0.92(0.75-1.10) |
|  |  |  |  |  |  |
| **DALYs (Disability-Adjusted Life Years)** |  |  |  |  |  |
| Global | 323798.59 ( 144342.15 - 675926.83 ) | 12.08 ( 5.38 - 25.21 ) | 607756.87 ( 272745.15 - 1268607.22 ) | 15.40 ( 6.91 - 32.13 ) | 0.72(0.68-0.75) |
| SDI |  |  |  |  |  |
| High SDI | 122087.07 ( 55322.29 - 254194.96 ) | 26.66 ( 12.08 - 55.34 ) | 154313.26 ( 70664.08 - 314967.06 ) | 31.37 ( 14.37 - 64 ) | 0.07(-0.09-0.24) |
| High-middle SDI | 61793.58 ( 27718.57 - 128454.50 ) | 11.05 ( 4.96 - 22.99 ) | 97184.50 ( 43255.09 - 205403.82 ) | 15.91 ( 7.10 - 33.31 ) | 1.21(1.17-1.25) |
| Middle SDI | 93350.16 ( 41377.04 - 195580.27 ) | 10.32 ( 4.57 - 21.76 ) | 214898.85 ( 95592.62 - 450661.83 ) | 17.26 ( 7.68 - 36.24 ) | 1.72(1.67-1.77) |
| Low-middle SDI | 36511.83 ( 15811.37 - 76977.48 ) | 6.59 ( 2.84 - 13.85 ) | 106509.68 ( 46621.43 - 223924.95 ) | 10.43 ( 4.56 - 21.89 ) | 1.60(1.56-1.64) |
| Low SDI | 9836.52 ( 4213.82 - 20814.37 ) | 4.35 ( 1.86 - 9.22 ) | 34425.20 ( 14799.71 - 72773.06 ) | 6.20 ( 2.67 - 13.05 ) | 1.21(1.19-1.24) |
| Health system |  |  |  |  |  |
| Advanced Health System | 153790.10 ( 70341.52 - 319860.56 ) | 22.81 ( 10.43 - 47.33 ) | 185952.38 ( 85392.44 - 382110.30 ) | 26.95 ( 12.31 - 55.17 ) | 0.17(0.04-0.31) |
| Basic Health System | 123699.30 ( 54098.23 - 260032.46 ) | 10.09 ( 4.40 - 21.23 ) | 266136.44 ( 118158.58 - 556135.21 ) | 17.19 ( 7.64 - 36.02 ) | 1.70(1.65-1.75) |
| Limited Health System | 43614.42 ( 18973.79 - 91859.01 ) | 5.9 ( 2.57 - 12.42 ) | 145769.44 ( 63565.22 - 306375.98 ) | 9.58 ( 4.17 - 20.11 ) | 1.76(1.69-1.83) |
| Minimal Health System | 2475.34 ( 1058.10 - 5180.47 ) | 4.02 ( 1.72 - 8.42 ) | 9473.22 ( 4080.63 - 20118.56 ) | 5.89 ( 2.56 - 12.41 ) | 1.21(1.13-1.30) |
| Regions |  |  |  |  |  |
| Andean Latin America | 4055.89 ( 1797.33 - 8748.39 ) | 20.98 ( 9.37 - 45.26 ) | 10129.25 ( 4423.90 - 21176.60 ) | 28.78 ( 12.57 - 60.15 ) | 1.06(0.98-1.13) |
| Australasia | 3873.53 ( 1763.71 - 8060.68 ) | 35.97 ( 16.34 - 74.76 ) | 6103.56 ( 2756.51 - 12632.80 ) | 41.77 ( 18.75 - 86.35 ) | 0.28(0.19-0.37) |
| Caribbean | 1979.83 ( 867.32 - 4095.22 ) | 10.50 ( 4.58 - 21.73 ) | 3161.50 ( 1381.53 - 6647.55 ) | 13.03 ( 5.7 - 27.37 ) | 0.76(0.69-0.82) |
| Central Asia | 1049.10 ( 438.14 - 2236.92 ) | 3.06 ( 1.28 - 6.52 ) | 2079.36 ( 886.90 - 4469.52 ) | 4.24 ( 1.81 - 9.1 ) | 1.17(1.10-1.24) |
| Central Europe | 1014.73 ( 425.90 - 2088.93 ) | 1.63 ( 0.68 - 3.36 ) | 1032.65 ( 442.86 - 2156.63 ) | 2.00 ( 0.86 - 4.17 ) | 0.63(0.58-0.68) |
| Central Latin America | 20163.69 ( 9003.77 - 42043.01 ) | 23.55 ( 10.46 - 49.22 ) | 35314.12 ( 15577.76 - 73735.75 ) | 25.74 ( 11.36 - 53.72 ) | -0.09(-0.25-0.06) |
| Central Sub-Saharan Africa | 958.26 ( 406.25 - 1975.20 ) | 3.83 ( 1.63 - 8.00 ) | 3861.86 ( 1673.68 - 7930.54 ) | 5.82 ( 2.51 - 12.02 ) | 1.28(1.12-1.43) |
| East Asia | 48225.91 ( 20818.56 - 100142.96 ) | 7.25 ( 3.13 - 15.07 ) | 89991.43 ( 39441.80 - 185701.88 ) | 13.38 ( 5.91 - 27.61 ) | 2.06(1.91-2.21) |
| Eastern Europe | 2097.69 ( 868.62 - 4466.82 ) | 1.83 ( 0.76 - 3.89 ) | 2323.67 ( 970.02 - 4890.37 ) | 2.35 ( 0.97 - 4.92 ) | 0.97(0.92-1.02) |
| Eastern Sub-Saharan Africa | 3903.29 ( 1645.09 - 8198.57 ) | 4.46 ( 1.88 - 9.40 ) | 12497.08 ( 5380.7 - 26310.19 ) | 5.77 ( 2.49 - 12.17 ) | 0.86(0.83-0.89) |
| High-income Asia Pacific | 38274.50 ( 16930.42 - 77128.81 ) | 41.4 ( 18.34 - 83.40 ) | 35520.50 ( 16037.31 - 72064.21 ) | 45.62 ( 20.81 - 92.93 ) | 0.25(0.20-0.30) |
| High-income North America | 39976.10 ( 17688.81 - 82638.29 ) | 26.6 ( 11.77 - 54.70 ) | 56162.19 ( 25718.34 - 113710.16 ) | 33.02 ( 15.20 - 66.90 ) | -0.53(-1.01--0.05) |
| North Africa and Middle East | 22386.47 ( 9812.91 - 47102.56 ) | 13.96 ( 6.10 - 29.29 ) | 59116.30 ( 26476.78 - 125706.81 ) | 18.4 ( 8.24 - 39.14 ) | 1.04(0.99-1.09) |
| Oceania | 352.20 ( 160.65 - 738.87 ) | 11.14 ( 5.06 - 23.3 ) | 1083.54 ( 471.33 - 2285.57 ) | 15.41 ( 6.71 - 32.47 ) | 0.82(0.65-0.98) |
| South Asia | 29342.13 ( 12824.11 - 62333.75 ) | 5.68 ( 2.49 - 12.07 ) | 98946.93 ( 43231.49 - 207059.23 ) | 9.94 ( 4.34 - 20.79 ) | 2.08(2.00-2.16) |
| Southeast Asia | 32853.77 ( 14429.10 - 66791.76 ) | 13.50 ( 5.92 - 27.49 ) | 92605.68 ( 41149.03 - 191024.08 ) | 25.06 ( 11.1 - 51.68 ) | 2.28(2.18-2.37) |
| Southern Latin America | 2627.83 ( 1179.43 - 5421.35 ) | 10.50 ( 4.71 - 21.66 ) | 5893.54 ( 2574.80 - 12247.50 ) | 16.74 ( 7.33 - 34.66 ) | 1.46(1.25-1.67) |
| Southern Sub-Saharan Africa | 2047.42 ( 881.84 - 4435.18 ) | 7.59 ( 3.27 - 16.39 ) | 4157.74 ( 1795.14 - 8746.01 ) | 9.47 ( 4.10 - 19.92 ) | 0.75(0.67-0.83) |
| Tropical Latin America | 4011.07 ( 1701.11 - 8427.30 ) | 4.97 ( 2.11 - 10.47 ) | 6557.65 ( 2825.05 - 13830.99 ) | 5.38 ( 2.33 - 11.29 ) | -0.16(-0.32-0.01) |
| Western Europe | 60788.92 ( 27584.87 - 126652.56 ) | 31.64 ( 14.36 - 65.86 ) | 66041.54 ( 29857.20 - 136985.13 ) | 35.16 ( 15.84 - 72.77 ) | 0.21(0.15-0.27) |
| Western Sub-Saharan Africa | 3816.23 ( 1628.96 - 8015.42 ) | 4.32 ( 1.85 - 9.10 ) | 15176.76 ( 6521.52 - 32235.88 ) | 6.24 ( 2.69 - 13.23 ) | 0.92(0.74-1.10) |
